# Integrated transcriptome and cell phenotype analysis suggest involvement of PARP1 cleavage, Hippo/Wnt, TGF-β and MAPK signaling pathways in ovarian cancer cells response to cannabis and PARP1 inhibitor treatment

**DOI:** 10.3389/fgene.2024.1333964

**Published:** 2024-01-23

**Authors:** Nurit Shalev, Michelle Kendall, Navin Kumar, Sudeep Tiwari, Seegehalli M. Anil, Hagit Hauschner, Savvemala G. Swamy, Adi Doron-Faingenboim, Eduard Belausov, Bruce E. Kendall, Hinanit Koltai

**Affiliations:** ^1^ The Mina and Everard Goodman Faculty of Life Sciences, Bar-Ilan University, Ramat Gan, Israel; ^2^ Volcani Center, Agriculture Research Organization, Institute of Plant Science, Rishon LeZion, Israel; ^3^ Canna Onc Research, Santa Barbara, CA, United States

**Keywords:** ovarian cancer, cannabis, phytocannabinoids, apoptosis, cell cycle, mesenchymal phenotype, endoplasmic reticulum stress, Hippo/Wnt pathway

## Abstract

**Introduction:** *Cannabis sativa* is utilized mainly for palliative care worldwide. Ovarian cancer (OC) is a lethal gynecologic cancer. A particular cannabis extract fraction ('F7′) and the Poly(ADP-Ribose) Polymerase 1 (PARP1) inhibitor niraparib act synergistically to promote OC cell apoptosis. Here we identified genetic pathways that are altered by the synergistic treatment in OC cell lines Caov3 and OVCAR3.

**Materials and methods:** Gene expression profiles were determined by RNA sequencing and quantitative PCR. Microscopy was used to determine actin arrangement, a scratch assay to determine cell migration and flow cytometry to determine apoptosis, cell cycle and aldehyde dehydrogenase (ALDH) activity. Western blotting was used to determine protein levels.

**Results:** Gene expression results suggested variations in gene expression between the two cell lines examined. Multiple genetic pathways, including Hippo/Wnt, TGF-β/Activin and MAPK were enriched with genes differentially expressed by niraparib and/or F7 treatments in both cell lines. Niraparib + F7 treatment led to cell cycle arrest and endoplasmic reticulum (ER) stress, inhibited cell migration, reduced the % of ALDH positive cells in the population and enhanced PARP1 cleavage.

**Conclusion:** The synergistic effect of the niraparib + F7 may result from the treatment affecting multiple genetic pathways involving cell death and reducing mesenchymal characteristics.

## 1 Introduction

Ovarian cancer (OC) is the most lethal gynecologic cancer with about 70% of patients are diagnosed in late stages, and late-stage OC is usually incurable (Cortez et al., 2018). Mortality is not reduced by population-level monitoring and no screening test for OC is routinely used (Menon et al., 2021). The standard of care includes cytoreductive surgery, followed by platinum-based chemotherapy (Barnett, 2016). Nevertheless, disease relapse in most of the cases after 24 months, and multi-drug resistance may develop.

*Cannabis sativa* is utilized worldwide for palliative care and to alleviate various symptoms associated with medical conditions (Corroon et al., 2019). Several dozen compounds are biosynthesized in the female inflorescence of each *C. sativa* strain. In total, around 600 different molecules can be found in cannabis, among them around 150 phytocannabinoids and hundreds of flavonoids and terpenes (Aizpurua-Olaizola et al., 2016; Hanuš et al., 2016; Gülck and Møller, 2020).

Multiple studies suggest that phytocannabinoids have anti-cancer properties. They inhibit several different features associated with cancer cells and tumors, including inhibiting cell proliferation and migration, inducing cell death, reducing angiogenesis, and inhibiting cancer cells’ invasiveness. This was demonstrated in several different cancer types, including cancers of the skin, lung, breast, prostate, and brain (Hinz and Ramer, 2019). The best-studied anti-cancer activity is that of the most common phytocannabinoids cannabidiol (CBD) and Δ9–tetrahydrocannabinol (THC), and related synthetic compounds (e.g., HU-210 and WIN-55 212-2) (Hinz and Ramer, 2019; Kovalchuk and Kovalchuk, 2020; Tomko et al., 2020).

Phytocannabinoids have been found to affect cancer cells and tumors via several different genetic pathways and molecular mechanisms. For example, several signal transduction pathways can be activated by phytocannabinoids to induce cancer cell death, including cell cycle arrest, endoplasmic reticulum (ER) stress, oxidative stress, autophagy and/or apoptosis (Hinz and Ramer, 2019; Kovalchuk and Kovalchuk, 2020; Tomko et al., 2020; Koltai and Shalev, 2022).

However, the effectiveness against OC of cannabis compounds has been examined in only a few studies. CBD was demonstrated to reduce proliferation in cell line of OC and in a model of chick embryo (i.e., in ovo) and to increase paclitaxel effectiveness *in vitro* and in ovo once administrated as a pre-treatment or in combination with paclitaxel (Fraguas-Sánchez et al., 2020a; Fraguas-Sánchez et al., 2020b). Treatment with Laetrile and ‘CBD oil’ that contains multiple molecules altered expression of genes in low-grade serous ovarian cancer in a single patient case-study (Barrie et al., 2019). In addition, it was shown *in vitro* and *in vivo* that CBD inhibits OC cell growth (Ma et al., 2023). Based on cell line studies it was suggested that it inhibits cell growth by disrupting the LAIR-1-mediated interference with PI3K/AKT/mTOR pathway and mitochondrial bioenergy metabolism (Ma et al., 2023).

Recently, we identified a high-THC cannabis-extract fractions and combinations of cannabis molecules that have cytotoxic activity against OC cells (Shalev et al., 2022). These extract fractions and compound-combinations induced cell apoptosis (Shalev et al., 2022). Moreover, the F7 fraction containing mostly THC, cannabichromene (CBC) to a lesser extent, with smaller proportions of cannabinol (CBN) and cannabigerol (CBG), and traces amount of other phytocannabinoids and terpenes (Peeri et al., 2021; Shalev et al., 2022), increased OC cell sensitivity to the poly(ADP-ribose)-polymerase (PARP)1 inhibitor niraparib *in vitro*. It was demonstrated that niraparib + F7 activity involves the wingless/int1 (Wnt) signaling pathway (Shalev et al., 2022).

To better characterize the signaling pathways that might be involved with the response of OC cells to niraparib + F7, in the current study we began with broad exploration of transcriptomic related changes in response to the F7 and/or niraparib treatments. We profiled gene expression following the treatments and examined cell phenotypes associated with apoptosis, including ER stress and cell cycle progression. In addition, phenotypes associated with mesenchymal properties were examined, including cell migration, actin arrangement and percentage of aldehyde dehydrogenase (ALDH) positive (+) cells in population. Finally, the level of PARP1 cleavage was evaluated with the niraparib + F7 treatment.

## 2 Materials and methods

### 2.1 Plant extraction

The dry inflorescence of *C. sativ*a strain Dairy Queen (DQ) (IMC, Israel), which is a high Δ9–tetrahydrocannabinol (THC) strain, was extracted as described previously (Shalev et al., 2022). The extract was decarboxylated by heating to 220°C for 10 min followed by dissolving and diluting to the desired concentration in methanol. The diluted extract was filtered through a 0.45 µm syringe filter (Shalev et al., 2022).

### 2.2 Extract fractionation

The complete decarboxylated crude extract was divided into fractions by using a flash chromatography apparatus (Flash Pure, Buchi, C-810) equipped with a diode array detector. The column used for separation was an Ecoflex C-18 80g, 50 µm spherical, max. Pressure 180 psi, the mobile phase was 80%–85% methanol in water with flow rate of 30 mL/min. Methanol was evaporated from each fraction using a rotary vacuum evaporator at 30°C and the remaining water was lyophilized. The dried fraction tubes of F7 (Shalev et al., 2022) were weighed and reconstituted with methanol to produce stock solution in concentrations of 2 mg/mL, and stored at −20°C.

### 2.3 Chemical analysis

HPLC (High performance liquid chromatography, 1260 Infinity II, Agilent) equipped with a Raptor ARC-18 for LC-UV column (150 mm × 4.6 mm ID, pore size 2.7 µm) was used to analyze phytocannabinoids content in each fraction as described previously (Peeri et al., 2021; Shalev et al., 2022). For chemical analysis of terpenes, 1 μL of each sample was analyzed by a gas chromatography-mass spectrometer (GC8860-MS5977B Agilent) equipped with 30 m, 0.25 mm ID, 5% cross-linked phenylmethyl siloxane capillary column (HP-5MS) with 0.25-μm film thickness, was used as described in (Peeri et al., 2021).

### 2.4 Cell culture

OC cell lines OVCAR3 (ATCC, HTB161; Adenocarcinoma) and Caov3 (ATCC, HTB75; Adenocarcinoma) were cultured in RPMI medium (01-100-1A, Biological Industries, Israel), supplemented with 20% fetal bovine serum (FBS) (04-127-1A, Biological Industries, Israel) and Dulbecco’s Modified Eagle Medium (DMEM) medium (01-055-1A, Biological Industries, Israel) supplemented with 10% FBS respectively. All media were supplemented with 1% Pen-Strep, 1% L-Glutamine and 0.02% plasmocin. Cells were incubated in 37°C in a humidified atmosphere, in environmental containing 5% CO_2_-95% air. Niraparib (AG0038ZU; Angene, China) was dissolved in DMSO to produce stock solution concentration of 2 mg/mL and diluted with growth medium in every experiment according to the desired concentration. DMSO and/or Methanol were used as a negative and vehicle control in the highest concentration treatments. Phosphate buffered saline (PBS) (DPBS, 02-023-1A, Biological Industries, Israel) was used for washing cells in all biological assays.

### 2.5 RNA sequencing and transcriptome analysis

1.5 × 10^6^ cell/well were seeded in 6-well plate in 2 mL medium per well for RNA preparation. After 24 h of incubation of cells in conditions described above, cells were treated with treatments or controls for 6 h. The cells were subsequently harvested, and total RNA was extracted using a TRI reagent (T9424, Sigma-Aldrich, United States) according to the manufacturer’s protocol. In parallel, cells were treated under the same conditions for 48 h and cell viability was determined as described in (Shalev et al., 2022), to verify the treatments effectiveness. The RNA was kept at −80°C until further analysis. Using the INCPM mRNA Seq protocol sequencing libraries were prepared. Sixty bp single reads were sequenced on one lane of an Illumina HiSeq. Transcriptome analysis was done as follows: a filtering and cleaning procedure was performed on the raw-reads. To trim read-end nucleotides with quality scores <30 FASTX Toolkit (http://hannonlab.cshl.edu/fastx_toolkit/index.html, version 0.0.13.2) was used. FASTQ Quality Filter was used to remove reads with less than 70% base pairs with a quality score ≤30. Using STAR software (v2.7.10; Dobin et al., 2012) clean-reads were mapped to the human genome (National Center for Biotechnology Information (NCBI); GRCh38; https://www.ncbi.nlm.nih.gov/genome/guide/human/). Cufflinks (v2.2) combined with gene annotations from the NCBI (Trapnell et al., 2010) was used for gene abundance estimation. Principal component analysis (PCA) and Heatmap visualization were carried out using R Bioconductor (Gentleman et al., 2004). Completion of differential expression analysis was done using the DESeq2 R package (Love et al., 2014). Genes considered differentially expressed if they varied in their expression from the control more than twofold, with an adjusted *p*-value of no more than 0.05 (Benjamini and Hochberg, 1995). Pathway analysis was done using the KEGG mapper tool (http://www.genome.jp/kegg/tool/map_pathway2.html). For pathway enrichment analysis the Enrichr tool was used (http://amp.pharm.mssm.edu/Enrichr/).

### 2.6 Quantitative real-time PCR

1.5 × 10^6^ cell/well were seeded in 6-well plate in 2 mL medium per well and incubated for 24 h prior treatment. Cells were treated with cannabis extract fraction and/or niraparib for 6, 9 or 24 h (as described in Shalev et al., 2022). TRI reagent (T9424, Sigma-Aldrich, United States) was used to extract RNA. RNA was reverse-transcribed according to manufacturer’s protocol (PB30.11-10, qPCRBIO). PCR was performed as described in (Shalev et al., 2022). The sequence of primers is in Supplementary Table S1.

### 2.7 Apoptosis assay

Caov3 and OVCAR3 cells were seeded in 6-well TC plates at a density of 5 × 10^5^ cells/well in 2 mL medium, 24 h before treatment. Treatment duration was 48 h and apoptosis assay was performed as described in (Shalev et al., 2022), using an MEBCYTO Apoptosis Kit with Annexin V-FITC and propidium iodide (PI) (4700; MBL MA, United States). Apoptosis rates were determined with flow cytometry.

### 2.8 Cell cycle analysis

Caov3 and OVCAR3 cells were seeded in 6-well TC plates at a density of 5 × 10^5^ cells/well in 2 mL medium, 24 h before treatment. Treatment duration was 24 h, followed by cell harvest with 250 μL trypsin for 5 min, adding 1 mL complete medium, and centrifuging for 10 min at 1800 rpm. The cell pellet was washed once with 1 mL of PBS, 70% cold ethanol was used for fixation followed by overnight incubation at 4°C. 1 mL of PBS was used to wash twice the fixed cells and then cells were stained with 500 μL of 20 μg/mL PI solution (AB-ab14083, Abcam) containing 50 μg/mL RNase A (EN0531, Thermo Scientific, UDA) for 30 min in the dark. Cell populations in the different phases of the cell cycle were determined with flow cytometry.

### 2.9 ALDH activity assay

The Aldefluor assay kit (1700, STEMCELL Technologies, Vancouver Canada) was used to assess ALDH activity in cells by flow cytometry (Wang et al., 2012). Caov3 and OVCAR3 cells were seeded in 6-well TC plates at a density of 1 × 10^6^ cells/well in 2 mL medium, 24 h before treatment. 48 h after treatments, single cells were harvested with trypsin followed by Aldefluor buffer wash, and 5 × 10^5^ cells/sample were incubated in Aldefluor buffer containing ALDH fluorescent substrate (2.5 μL/mL) at 37°C for 40 min, while one sample was treated with 2.5 μL/mL of diethylaminobenzaldehyde (DEAB, an ALDH inhibitor) immediately after ALDH substrate addition, as a negative control. After incubation, cells were washed once with cold Aldefluor assay buffer, and kept on ice. The ALDH-expressing cells (ALDH+) were analyzed with flow cytometry.

### 2.10 Flow cytometry analysis

Flow cytometry LSR-FORTESSA (BD, United States) was utilized to analyze apoptosis, cell cycle and ALDH + cells. Cells were determined to be apoptotic if they were Annexin V+/PI- (early apoptosis) or Annexin V+/PI+ (late apoptosis). Cells were considered live when defined as Annexin V-/PI-, and necrotic when defined as Annexin V-/PI+. For cell cycle, cell count versus linear fluorescence excitation light at 610 nm is used to create a histogram of the DNA content distribution across the phases of the cell cycle. At least 10,000 cells per sample were examined for each specimen. For ALDH, a fluorescence channel at 488 nm vs. SSC dot plot was created, while the DEAB control was used to confirm gating areas. At least 20,000 cells per sample were examined for each specimen. Data analysis was preformed using FlowJo software (FlowJo, V 10.8.1, BD Biosciences, CA, United States).

### 2.11 Scratch-wound assay

Cells were seeded into a 96-well plate at a density of 5 × 10^4^ per well for Caov3 and 1 × 10^5^ for OVCAR3 in 100 µL of medium. After 24 h, wells were scratched perpendicularly with a 200 µL tip to produce a cell-free area followed by wash with 100 µL PBS. Treatment solution in volume of 100 µL were applied. After scratching photos were taken at 0, 24, 30, and 48 h for Caov3 and 0, 48, 72 and 96 h for OVCAR3. The scratch area was calculated using ImageJ (version 1.53a) as percent of scratch area at time x in relation to time 0:

$$\frac{\left(\;\text{cell}\;\text{free}\;\text{area}\;\text{at}\;\text{time}\;x\right)\times 100\;}{\left(\;\text{cell}\;\text{free}\;\text{area}\;\text{at}\;\text{time}\;0\right)}$$

### 2.12 Cytoskeleton staining

OVCAR3 cell were seeded on glass bottom culture dishes at a density of 5 × 10^4^ per plate. 3 days later, cells were induced for stress as described in (Shalev et al., 2022), in induction medium contains RPMI with 5% FBS and recombinant Human IL-1β (200-01B-10, Pepro Tech, NJ, United States) in concentration of 20 ng/mL. Treatments were given at under-lethal concentrations after 24 h of induction, for 16 h. For nuclear and F-actin staining, cells were washed with PBS, formaldehyde solution 3.7% in PBS for 10 min was used for fixation, Triton™ X-100 0.1% (T8787; Sigma-Aldrich, MO, United States) for 5 min was used for permeabilization, Bovine Serum Albumin 1% (BSA; A7284; Sigma-Aldrich, MO, United States) for 30 min at room temperature was used for blocking. The cells were labeled with F-ActinGreen 488 (AP-FP031, ABP Biosciences, MD, United States) for 30 min and Hoechst (AP-FP027, ABP Biosciences, MD, United States) for 15 min. Image acquisition was based on at least 10 optical sections and was done using a Leica SP8 laser scanning microscope (Wetzlar, Germany), equipped with a 405 and 552 nm solid state lasers, HCX PL APO CS 10×/0.40 or HC PL APO CS 63×/1.2 water immersion objectives (Leica, Wetzlar, Germany) and Leica Application Suite X software (Wetzlar, Germany). PMT and HyD (hybrid) detectors were used for detection of Hoechst and F-actin emission signals in ranges of 415–490 and 565–660 nm, respectively. Experiments were repeated 4 times and at least 3 images were captured from each slide. Signals of 10 cells from 3 pictures for each treatment and control were analyzed using ImageJ (version 1.53a). Pixels with green signal intensity above a threshold of 50 were counted along the cell diameter. Dark or dimmed cells (mean intensity<25) were not measured.

### 2.13 Western blotting

OVCAR3 or Caov3 (2 × 10^6^ cells each) were grown in 6 well culture plates. All culture medium was removed and washed with 1 mL PBS for cell lysis. Lysates were prepared in 200 µL 1X RIPA buffer (Cat.# 20-188, Merck, Darmstadt, Germany), containing 1X Halt Protease Inhibitor Cocktail (Cat.# 78429, Thermo Fisher Scientific, Massachusetts, United States) at 4°C for 30 min. Cell lysates were mixed with 2X Laemmli sample buffer (Bio-Rad, California, United States) in a 1:1 ratio and heated for 10 min at 95°C. Cell lysates were resolved on 12.5% SDS-polyacrylamide gels. Proteins were transferred onto nitrocellulose membranes through the semi-dry method using turbo transfer system (Cat. # 1704150, Bio-Rad, California, United States). Membranes were blocked in 5% non-fat skimmed milk, dissolved in 1X TBST buffer and kept for 1 h at room temperature. The membranes were probed with a primary Anti-PARP1 antibody (Rabbit monoclonal, Cat. #E102, ab32138, Abcam, Cambridge, United Kingdom) (1:1000 dilution) overnight at 4 °C. After overnight incubation, membranes were washed with 1X TBST buffer twice for 5 min each time. Further, membranes were probed with secondary Goat Anti-Rabbit IgG (H + L)-HRP Conjugate antibody (1: 3000 dilution) for 1 h at room temperature. Spot intensity was visualized using SuperSignal™ West Femto Maximum Sensitivity Substrate (Cat. # 34094, Thermo Fisher Scientific, Massachusetts, United States) and imaged with Fusion Pulse 6 (Vilber, France). Spot intensities were quantified using the software ImageJ.

### 2.14 Statistical analysis

Mean ± standard error (SE) of replicate analyses are presented; number of independent experiments (n) is indicated in each set of results. Two-way ANOVA was used to determine the effect of treatment, time and their interaction. When the interaction effect was significant treatments were compared at each time-point as above. For statistical analysis we used the JMP 16 package (SAS Inc, NC, United States; https://www.jmp.com/en_us/home.html).

## 3 Results and discussion

### 3.1 Gene expression profile of F7 and/or niraparib treatments of OC cell lines

**FIGURE 1:**
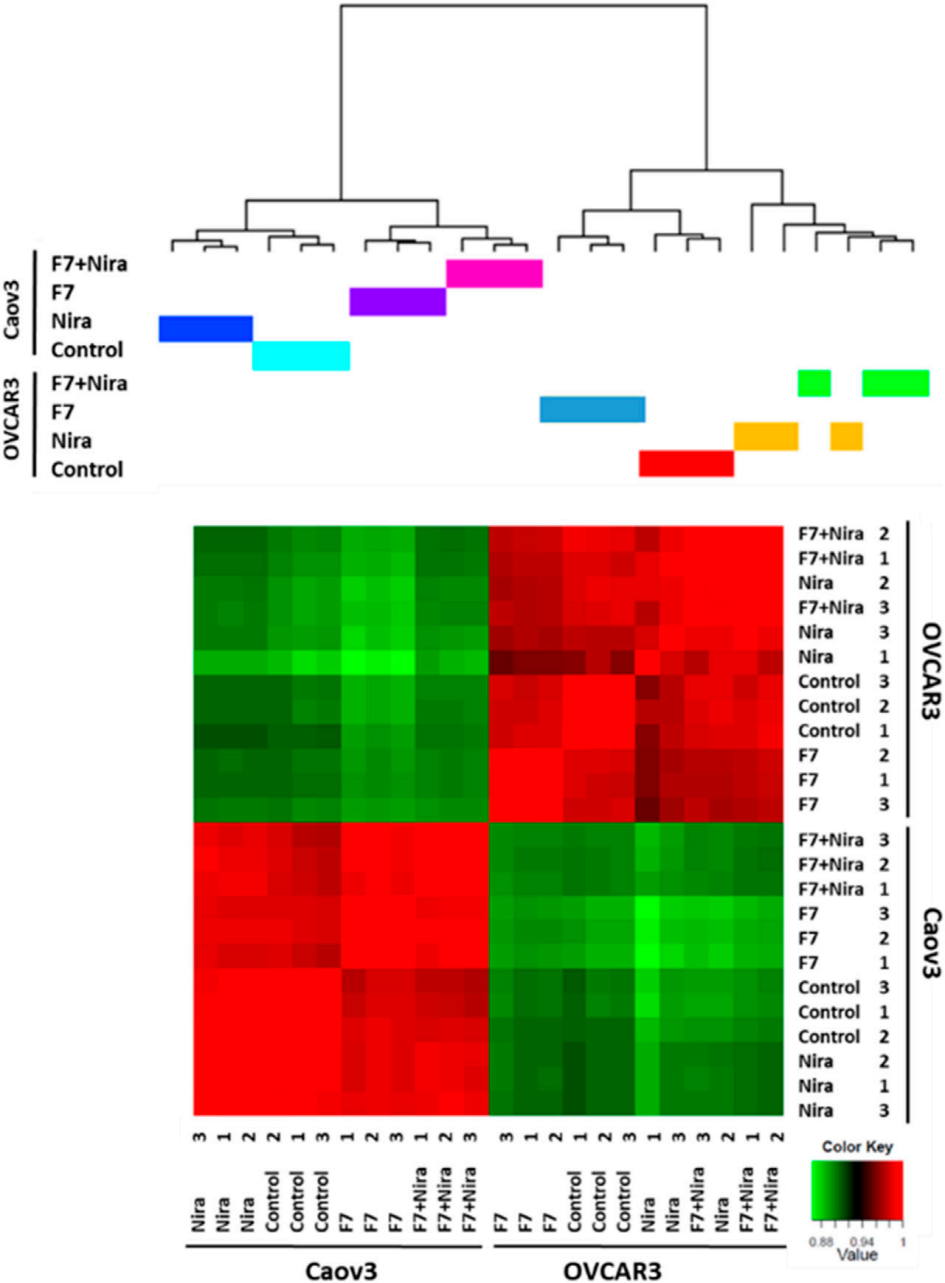
Hierarchical clustering of all samples based on the identified genes in Caov3 and OVCAR3 cells treated with niraparib (Nira), F7 or the F7+niraparib combination. Vehicle control is “control.” The last digit indicates the replicate number (1-3). Hierarchical clustering was calculated using Pearson correlations out of dissimilarity tables, among the four conditions based on genes expression [counts per million (CPM)] followed by a log2 transform. Colors indicate the correlation values calculated by Pearson correlations with R software. Correlation ranges from −1 to 1, where −1 indicates anti-correlation (or negative correlation), 0 no correlation and 1 correlation (or positive correlation).

Previously we showed that fraction F7 of the DQ *C. sativa* extract interacts synergistically with niraparib for cytotoxicity in OC cell lines (Shalev et al., 2022). Composition of F7 was previously described (Peeri et al., 2021; Shalev et al., 2022). Niraparib is a PARP inhibitors being introduced in clinical practice for OC patients in the setting of maintenance treatment following platinum-based chemotherapy (Caruso et al., 2017; Scott, 2017). To determine the effect of niraparib + F7 on gene expression profile in comparison to niraparib or F7, we used RNA sequencing. The RNAseq analysis of collected samples provided 424,558,760 high-quality reads. For each sample, ∼98% of the reads were mapped to the human genome reference. Each of the two cell lines cluster separately based on the profile of gene expression (Figure 1), suggesting that Caov3 and OVCAR3 are different in their expression profile in both control and in response to the F7 and/or niraparib treatments. In Caov3, control and niraparib treatments are clustered together while F7 and F7+niraparib are clustered together separately from niraparib or control (Figure 1). In contrast, in OVCAR3, clustering results show that the control and F7 treatments are clustered together while niraparib and F7+niraparib are clustered together and separately from the control and F7 (Figure 1). Similar distribution of the expression data is evident also by PCA analysis (Supplementary Figure S1A). Comparing the gene expression of niraparib + F7 treatments vs control using Volcano plot and MA analysis (log2 fold change > 1 and Padj < 0.05 significance), 1668 differentially expressed genes, including 823 upregulated genes and 845 downregulated genes were detected in Caov3. In OVCAR3, 1054 differentially expressed genes, including 442 upregulated genes and 612 downregulated genes were detected (Supplementary Figures S1B, C).

Accordingly, we have examined clustering results of the gene expression profile for the 500 genes that are differentially expressed. Here too the Caov3 and OVCAR3 expression profiles are primarily different from each other when treated with the control and in response to the F7 and/or niraparib treatments (Supplementary Figure S2). In Caov3, control and niraparib treatments cluster together and those of F7 and F7+niraparib cluster together, separately from niraparib and control (Supplementary Figure S2). In contrast, in OVCAR3, the results show F7 treatment clusters separately while the vehicle control, niraparib and F7+niraparib cluster together and separately from F7 (Supplementary Figure S2). The RNA-Seq data were deposited in the NCBI sequence read archive (SRA) as indicated below.

These results suggest that there are considerable differences between these two cell lines in their response to the F7 and/or niraparib treatments. These cell lines were isolated from two different high-grade serous ovarian carcinoma patients, and differ in their genetic background (https://www.atcc.org). As might be expected, Caov3 and OVCAR3 are different in some aspects in relation to e.g., expression of genes associated with the canonical Wnt signaling pathway and in their gene mutations (Beaufort et al., 2014). These differences may account for the separate clustering of gene expression results between the two cell lines.

### 3.2 Biological processes that are differentially expressed during the treatment by niraparib + F7 in each or both cell lines

An examination of the annotations for the differentially regulated genes expressed due to the synergistic treatments of niraparib + F7 in Caov3 or OVCAR3 cells suggest that there are several genetic pathways significantly enriched. The biological processes that are significantly enriched with annotated genes (ratio of no. genes upregulated or downregulated/no. of genes in the pathway) and were upregulated or downregulated by the F7+niraparib treatment vs vehicle control in the two cell lines are in Supplementary Tables S2–S7.

In Tables 1, 2 the biological process (with KEGG pathways ID) that are significantly enriched with annotated genes that are differentially expressed, either upregulated or downregulated (Tables 1, 2, respectively) only by the niraparib + F7 treatment and in both OVCAR3 and Caov3 cell lines.

**TABLE 1:** Biological processes (with KEGG pathway IDs) that are significantly enriched (ratio of no. genes upregulated/no. of genes in the pathway ≥ 0.02; *p* ≤ 0.05) with annotated genes that are upregulated specifically by the F7+niraparib treatment in both OVCAR3 and Caov3 cell lines.

Pathway	KEGG ID	Differentially expressed genes number	Number of genes in the pathway	*p*-value	Corrected *p*-value	Enrichment
IL-17 signaling pathway	hsa04657	6	93	9.34E-08	7.09E-06	0.064516
Parathyroid hormone synthesis, secretion and action	hsa04928	6	106	1.95E-07	1.28E-05	0.056604
TNF signaling pathway	hsa04668	5	112	6.50E-06	0.000267	0.044643
Human T-cell leukemia virus 1 infection	hsa05166	6	219	1.13E-05	0.000444	0.027397
Amphetamine addiction	hsa05031	4	68	2.05E-05	0.000749	0.058824
Hepatitis B	hsa05161	5	163	3.73E-05	0.001055	0.030675
MAPK signaling pathway	hsa04010	6	295	5.74E-05	0.001452	0.020339
Prostate cancer	hsa05215	4	97	7.74E-05	0.00191	0.041237
Ferroptosis	hsa04216	3	40	0.000121	0.002541	0.075
Transcriptional misregulation in cancer	hsa05202	4	186	0.000859	0.012112	0.021505
Insulin resistance	hsa04931	3	108	0.001952	0.022399	0.027778
HIF-1 signaling pathway	hsa04066	3	109	0.002002	0.022412	0.027523
Osteoclast differentiation	hsa04380	3	128	0.003121	0.02962	0.023438
Dopaminergic synapse	hsa04728	3	131	0.003326	0.030972	0.022901
Apoptosis	hsa04210	3	136	0.003686	0.033145	0.022059
Cocaine addiction	hsa05030	2	49	0.005748	0.043978	0.040816
Mineral absorption	hsa04978	2	53	0.006656	0.049771	0.037736

**TABLE 2:** Biological processes (with KEGG pathway IDs) that are significantly enriched (ratio of no. genes downregulated/no. of genes in the pathway ≥0.02; *p* ≤ 0.08) with annotated genes that were downregulated specifically by the F7+niraparib treatment in both OVCAR3 and Caov3 cell lines.

Pathway	KEGG ID	Differentially expressed genes number	Number of genes in the pathway	*p*-value	Corrected *p*-value	Enrichment
Basal cell carcinoma	hsa05217	4	63	0.000268	0.010775	0.063492
Signaling pathways regulating pluripotency of stem cells	hsa04550	6	140	6.33E-05	0.003625	0.042857
Hippo signaling pathway	hsa04390	5	154	0.000889	0.0228	0.032468
TGF-beta signaling pathway	hsa04350	3	94	0.010386	0.084614	0.031915
Hepatitis C	hsa05160	4	155	0.006466	0.063156	0.025806
Wnt signaling pathway	hsa04310	4	160	0.007199	0.067622	0.025

*p* ≤ 0.08 was chosen for the Corrected *p*-value to include pathways with above 0.02 proportion of enrichment for significantly regulated genes.

### 3.3 Selected gene expression in biological processes enriched with annotated genes that were significantly regulated only by the F7+niraparib treatments vs control in both cell lines

In the pathways that are significantly enriched with annotated genes in the F7+niraparib treatment vs control in both examined cell lines (Tables 1, 2), we allocated genes that have similar tendency of expression in both cell lines.

In agreement with our previous findings (Shalev et al., 2022), the Hippo/Wnt signaling pathway (hsa04390/hsa04310 in Supplementary Figure S3) is involved in F7+niraparib synergy. In addition, number of genes have been altered and were similarly regulated in both cell lines following the combined niraparib + F7 treatment, including the upregulated gene *Amphiregulin* (*AREG*), and a number of downregulated genes including *Autocrine bone morphogenetic protein-4* (*BMP4*), *AJUBA* and *Inhibitor of DNA binding* (*ID*)1 and *ID2* (arrows in Supplementary Figure S3). The expression pattern of those genes was verified by qPCR (Supplementary Figures S3C–G for Caov3 and Supplementary Figures S3H–L for OVCAR3). The canonical Wnt pathway is aberrantly activated in various cancers and has a critical role in OC development (Arend et al., 2013; Nguyen et al., 2019). This pathway is associated with chemotherapy resistance in cancer with epithelial–mesenchymal transition (EMT) (Nguyen et al., 2019; Patel et al., 2019; Kaur et al., 2021). During EMT cells lose polarity and gain increased motility (Patel et al., 2019). BMP4 acts to increase mesenchymal state at least partially via ID proteins, highly conserved transcription regulators. *ID1* is a direct BMP target gene, and its expression can be upregulated by BMPs (Katagiri et al., 2002).

ID proteins are overexpressed in many cancer types and promote cancer initiation, progression and drug resistance (Ruzinova and Benezra, 2003; Lasorella et al., 2014). In our study, in Caov3, F7 and niraparib treatments reduced *ID1* gene expression to some extent, in accordance with the reduction in *BMP4* gene expression by the niraparib and F7 treatments. However, *ID1* expression was even further reduced by the combined niraparib + F7 treatment. In OVCAR3, F7 induced *ID1* gene expression but the combined F7+niraparib treatment reduced it to levels below those of niraparib treatment only. *ID2* gene expression was also substantially reduced by the F7 (in Caov3) and combined treatment (in both cell lines). *ID2* gene overexpression in the OC cell line SKOV-3 increased the cells’ invasive potential (Meng et al., 2009). ID proteins were shown to control the cell cycle by repressing expression of *INK4a* (P16), a cyclin-dependent kinase inhibitor (Ruzinova and Benezra, 2003). Although gene expression of *INK4a* was not changed considerably with the treatments in this study (Supplementary Figure S8), overexpression, or repression of other cell cycle regulators, as described above, might have led to cell cycle arrest exhibited here.

Another Hippo signaling related protein is AJUBA, a LIM domain protein, which is involved in various biological functions. In colon cancer, cells depleted of AJUBA were less proliferative and migrated less (Dommann et al., 2020). Here, *AJUBA* gene expression was reduced by all treatments in both cell lines, and especially by the combined treatment.

To conclude, it might be that F7 and/or niraparib affect multiple components of the Hippo pathway, including autocrine signals, to repress tumorigenicity of OC cell lines. However, *AREG*, another Hippo pathway component (Tung et al., 2017), was upregulated in its gene expression by all treatments in our study. An AREG-mediated increase in drug resistance of OC cell lines towards docetaxel and carboplatin was recorded, as well as overexpression of AREG in OC sphere cells (Tung et al., 2017). In OC patients, AREG is suggested to be derived from senescent stromal cells, and to be highly abundant in abdominal fluids of advanced OC patients and high AREG also correlates with poor prognosis of patients expressing wildtype TP53 (Lindzen et al., 2021).

In the Interleukin (IL)17 signaling pathway (hsa04657; Supplementary Figure S4A, B), *TNFAIP3* (A20) was upregulated in both cell lines following all examined treatments and as verified in qPCR (Supplementary Figure S4C for Caov3 and Supplementary Figure S4D for OVCAR3). A20 is an anti-inflammatory molecule that inhibits NF-κB activation (Heyninck and Beyaert, 2005). A20 acts as an oncogene in gastric cancers, breast cancers, acute lymphoblastic leukemia and melanoma cells but plays antitumor roles in colorectal carcinomas, B cell lymphomas and hepatocellular carcinomas (Chen et al., 2015; Shi et al., 2021).

In the transforming growth factor-β (TGF- β)/Activin signaling pathway (hsa04350; Supplementary Figure S5A, B) a number of genes were similarly regulated by niraparib + F7 treatment in both cell lines. In addition to *BMP4* and *ID* genes, expression of *Paired-like homeodomain 2* (*PITX2*) was downregulated in both cell lines (arrow in Supplementary Figure S5). Reduction in expression pattern of this gene was verified by qPCR (Supplementary Figure S5C for Caov3 and Supplementary Figure S5D for OVCAR3). Increased expression of *PITX2* was found in OC cells, and it was suggested to be involved in OC progression via promoting cell growth, migration and invasion, and tumor growth *in vivo* (Zhang et al., 2013).

In the signaling pathways regulating pluripotency of stem cells/MAPK signaling (hsa04550; Supplementary Figure S6A, B), a number of genes were similarly regulated in both cell lines. The downregulation of *Orthodenticle homeobox 1* (*OTX1*) and *Fibroblast growth factor receptor 2* (*FGFR2*; arrows in Supplementary Figures S6A, B) was verified by qPCR in both cell lines Supplementary Figures S6C, D for Caov3 and Supplementary Figures S6E, F for OVCAR3). Inhibition of FGFR2 increased cisplatin sensitivity in OC and FGFR2 expression silencing also inhibited proliferation of OC cells and reduced growth rates of ovarian tumor xenografts to some extent (Cole et al., 2010). FGFR2-related signaling was also demonstrated to induce cell migration and invasion in human pancreatic cancer (Nomura et al., 2008) and gastric cancer (Huang et al., 2017). *FGFR2* gene expression was repressed by the combined treatment in Caov3 in particular.

Downstream to MAPK signaling and FGFR signaling (hsa04550) is OTX1, a bicoid-like homeodomain transcription factor (Zhou et al., 2022). Expression of *OTX1* was significantly upregulated in cervical cancer tissue and cells, promoting cell proliferation, migration, and invasion (Zhou et al., 2022). It was suggested that by activating the Wnt signaling pathway, OTX1 promoted the progression of cervical cancer (Zhou et al., 2022). *OTX1* gene expression was repressed by F7 or the combined treatment in both cell lines.

In conclusion, F7 and/or niraparib + F7 treatments act in most cases to reduce expression of oncogenic genes from the Hippo/Wnt, TGF-β and MAPK Signaling Pathways.

### 3.4 Bioassays for the effectiveness of the treatments on activities related to the selected genes and pathways

#### 3.4.1 Determining treatment effects on cell apoptosis and ER stress

**FIGURE 2:**
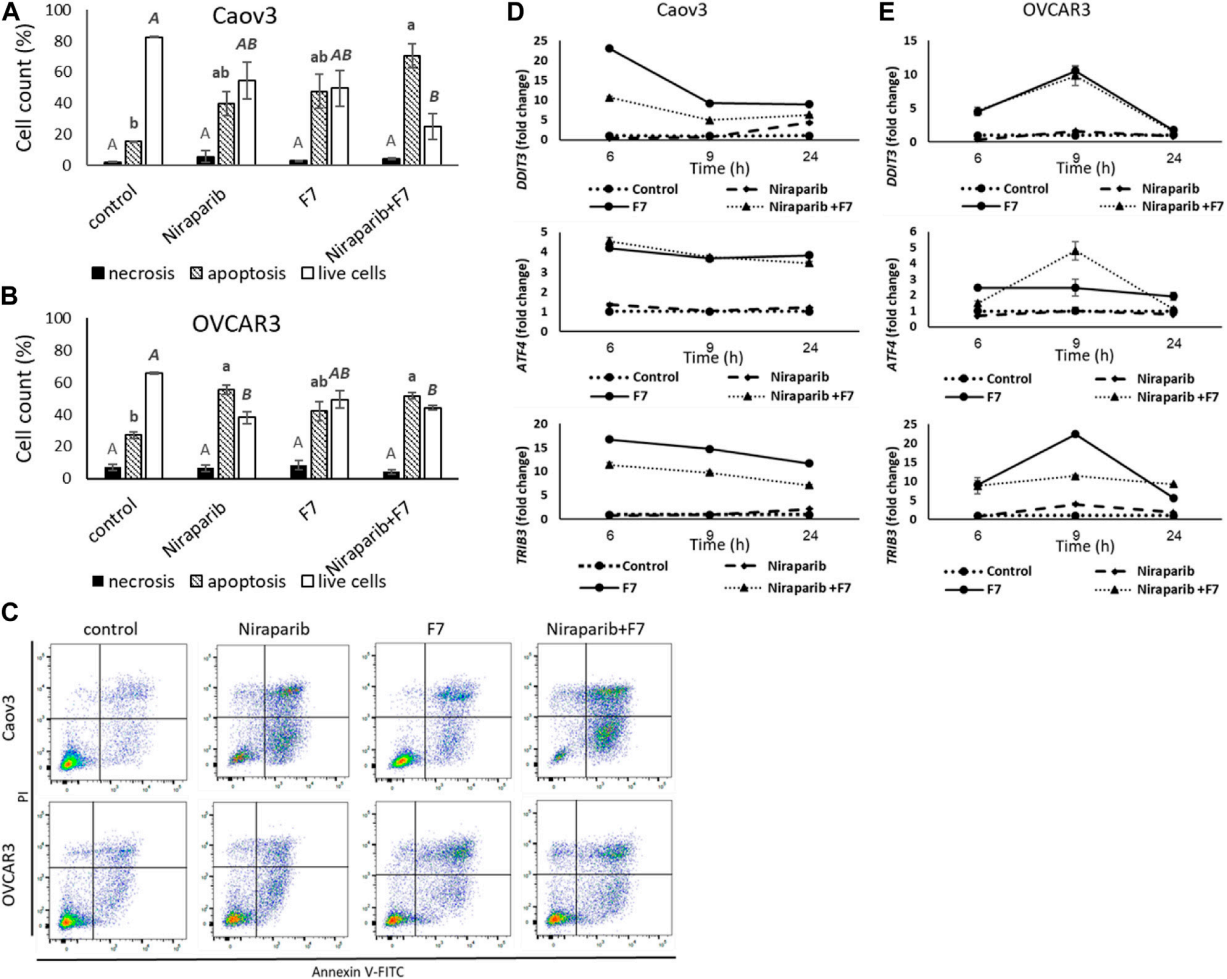
Percentage of necrotic, apoptotic or live cells of Caov3 **(A)** or OVCAR3 **(B)** cell lines were analyzed in FACS following treatment with control, niraparib (6 or 25 μg/mL for Caov3 or OVCAR3, respectively), F7 (17 or 24.5 μg/mL for Caov3 or OVCAR3, respectively), or niraparib + F7 at the corresponding concentrations, for 48 h. Doses of treatments are based on (Shalev et al., 2022). Control is vehicle control (0.25% DMSO+0.85% methanol v/v for Caov3 and 1.25% DMSO+1.23% methanol v/v for OVCAR3). Cells were stained with annexin V-FITC/PI, and 1× 10^4^ cells per treatment were analyzed. Error bars indicate ± standard error (*n* = 3). One-way ANOVA was performed and means without a common letter with similar font and style for necrotic, apoptotic or live cells denote expression levels that are significantly different by the Tukey-Kramer honest significant difference (HSD; *p* ≤ 0.05). Representative flow cytometry dot plots of Caov3 and OVCAR3 cells for Annexin V-PI counterstain **(C)**. Cells were gated according to unstained cells for analyses. Data analysis was preformed using FlowJo software (FlowJo, V 10.8.1, BD Biosciences, CA, United States). mRNA steady state level based on quantitative PCR in Caov3 **(D)** and OVCAR3 **(E)** cell lines treated for 6, 9 and 24 h with niraparib (6 or 25 μg/mL for Caov3 or OVCAR3, respectively), F7 (17 or 24.5 μg/mL for Caov3 or OVCAR3, respectively), or a combination of niraparib + F7 at the corresponding concentrations, relative to control. Quantitative PCR was used to determine gene transcript values as a difference between the target genes and a reference gene (HPRT) using the 2^−ΔΔCT^ method. Control is the vehicle control (0.3% DMSO+0.87% v/v methanol for Caov3 and 1.25% DMSO+1% v/v methanol for OVCAR3). Error bars indicate ± standard error (*n* = 3). The significance of the treatments is noted in Supplementary Table S8, since the “treatment” x “time” interaction was found to be significant by two-way ANOVA (*p* ≤ 0.05), one-way ANOVA was performed for each time-point separately. Means without a common letter denote expression levels that are significantly different by the Tukey-Kramer honest significant difference (HSD; *p* ≤ 0.05).

Previously we have demonstrated that the niraparib or F7 treatments lead to cell apoptosis (Shalev et al., 2022). Here we demonstrate that the combined treatment of niraparib + F7 leads to cell apoptosis (Figures 2A–C): 73.0% of apoptotic cells were recorded in the treated Caov3 population (only 16.7% of apoptotic cells in control; Figure 2A) and 51.7% were recorded in the treated OVCAR3 population (27.2% of apoptotic cells in control; Figure 2B). No changes were demonstrated in cell necrosis by the treatments in both cell lines (Figures 2A, B).

In the apoptosis pathway (hsa04210; Supplementary Figure S7), ER stress-related genes, including *Tribbles pseudokinase 3* (*TRIB3*), *DNA damage inducible transcript 3* (*DDIT3*; *CHOP*) and *Activating transcription factor 4* (*ATF4*) were induced by the treatments (Figure 2D, E; Supplementary Table S8). The Induction of *DDIT3* gene expression was evident mostly by F7 treatment and the combined niraparib + F7 treatment at 6 and 9 h in both cell lines (Figure 2D, E). *ATF4* and *TRIB3* transcription was induced mostly by the F7 and the combined niraparib + F7 treatments at all examined time points for Caov3 and at 9 h for OVCAR3 (Figure 2D, E). Since ER stress may lead to apoptotic cell death (Tabas and Ron, 2011), these results suggest that apoptosis may have resulted, at least partially, from ER stress induced by the F7 or niraparib + F7 treatments. In agreement, in other studies, it was shown that phytocannabinoids often induce ER stress in cancer cells followed by apoptosis (Mangal et al., 2021).

#### 3.4.2 Determining treatment effects on cell cycle arrest and on gene expression of cell cycle pathways

**FIGURE 3:**
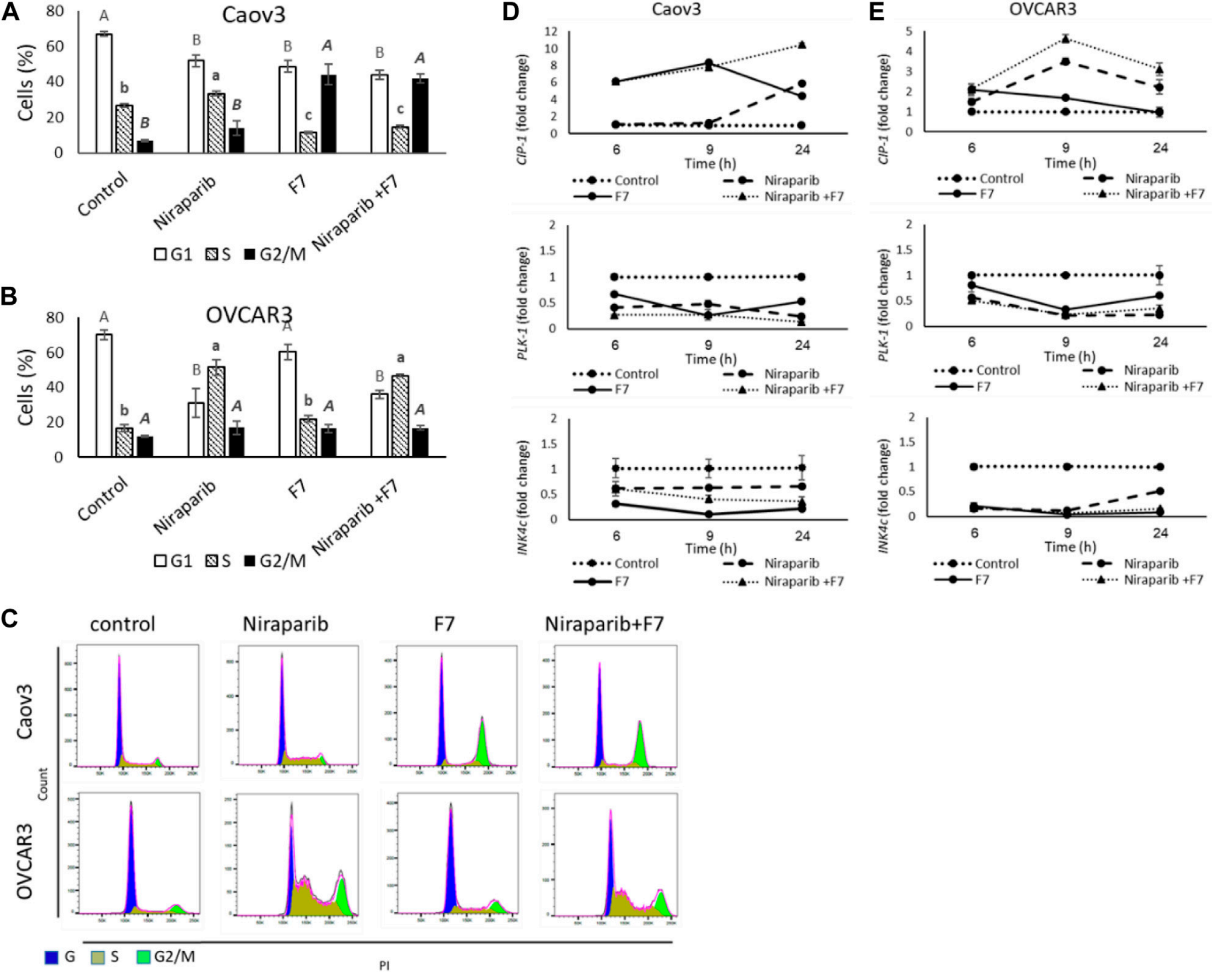
Percentage of cells in G1, S or G2/M phase of Caov3 **(A)** and OVCAR3 **(B)** cell lines were analyzed in FACS following treatments with niraparib (6 or 25 μg/mL for Caov3 or OVCAR3, respectively), F7 (19.4 or 20 μg/mL for Caov3 or OVCAR3, respectively) and niraparib + F7 at the corresponding concentrations, for 24 h. Doses of treatments are based on (Shalev et al., 2022). Control is vehicle control (0.3% DMSO+1% v/v methanol for Caov3 and 1.25% DMSO+1% methanol v/v for OVCAR3). Cells were stained with PI staining and 1× 10^4^ cells per treatment were analyzed. Error bars indicate ± standard error (*n* = 3). One-way ANOVA was performed and means without a common letter with similar font and style for G1, S or G2/M phase denote expression levels that are significantly different by the Tukey-Kramer honest significant difference (HSD; *p* ≤ 0.05). Representative flow cytometry dot plots of Caov3 and OVCAR3 cells for PI counterstain **(C)**. Cells were gated according to unstained cells for analyses. Data analysis was preformed using FlowJo software (FlowJo, V 10.8.1, BD Biosciences, CA, United States). mRNA steady state level based on quantitative PCR in Caov3 **(D)** and OVCAR3 **(E)** cell lines treated for 6, 9 and 24 h with niraparib (6 or 25 μg/mL for Caov3 or OVCAR3, respectively), F7 (17 or 24.5 μg/mL for Caov3 or OVCAR3, respectively), or a combination of niraparib + F7 at the corresponding concentrations, relative to control. Quantitative PCR was used to determine gene transcript values as a difference between the target genes and a reference gene (HPRT) using the 2^−ΔΔCT^ method. Control is the vehicle control (0.3% DMSO+0.87% v/v methanol for Caov3 and 1.25% DMSO+1% v/v methanol for OVCAR3). Error bars indicate ± standard error (*n* = 3). The significance of the treatments is noted in Supplementary Table S9, since the “treatment” x “time” interaction was found to be significant by two-way ANOVA (*p* ≤ 0.05), one-way ANOVA was performed for each time-point separately. Means without a common letter denote expression levels that are significantly different by the Tukey-Kramer honest significant difference (HSD; *p* ≤ 0.05).

Treatment of Caov3 with niraparib led to a minor increase in proportion of cells in G2/M phase of the cell cycle (14.1%) in comparison to vehicle control (6.9%; Figure 3A). The proportion of cells in S phase was also slightly increased (33.5% vs. 26.6% in niraparib and control, respectively; Figure 3A). However, treatment with F7 substantially increased proportion of cells in the G2/M phase (44.1%; Figure 3A). The combined niraparib + F7 treatment, similarly to F7, led to a marked increase in the G2/M phase of cell cycle (42.0%; Figure 3A). Treatment of OVCAR3 with niraparib and the combined treatment increased the proportion of cells in the S phase of the cell cycle (51.5% and 46.9%, respectively; Figure 3B) in comparison to the control (16.8%; Figure 3B). Representative flow cytometry dot plots of Caov3 and OVCAR3 cells for PI counterstain are presented in Figure 3C.

In accordance, the expression of the cell cycle pathway genes was significantly affected by the treatments and their duration (Figure 3D, E; Supplementary Table S9). *Cyclin dependent kinase inhibitor 1A* (*CIP1*; P21) was upregulated by F7 treatment mainly at 6 and 9 h in Caov3 and to a lesser extent at the same times in OVCAR3 (Figure 3D, E). The combined treatment induced *CIP1* at all examined time points in both cell lines (Figure 3D, E). Treatment with niraparib induced *CIP1* expression mainly at 24 h in Caov3 and at 9 and 24 h in OVCAR3 (Figure 3D, E). The *CIP* gene family can inhibit the activity of all cyclin-dependent kinases (CDKs) (Sherr and Roberts, 1995). Increase in *CIP1* (P21) expression leads to cell cycle arrest, in both p53 and p53-independent ways (Kreis et al., 2019). Increased expression of *CIP1* was associated mainly with G2/M arrest in several cancer types, including non-small-cell lung and endometrial cancer cells (Zhou et al., 2016; Pai et al., 2021). In addition, higher P21 expression in early-stage OC tumors is associated with no recurrence of tumor and low P21 expression is associated with reduced survival in older OC patients (Anttila et al., 1999; Schmider et al., 2000; Dall’Acqua et al., 2021).

Expression of *Polo like kinase 1* (*PLK1*) was downregulated substantially by all treatments at all examined time points in Caov3 and in OVCAR3 (Figure 3D, E). PLK1 facilitates progression from the G2 phase by promoting checkpoint recovery and mitotic entry in DNA damage-induced arrest in mammalian cells but not in unperturbed cell cycles (Zou and Lin, 2021). In p53-null cancer cells, such as Caov3 and OVCAR3 (Beaufort et al., 2014), depletion of PLK1 induced the activation of DNA damage checkpoint and promoted G2/M arrest and apoptosis (Jung et al., 2021). Expression of Inhibitors of CDK (*INK*)*4c* (P18) was downregulated by all treatments at all examined time points in both cell lines, and mainly in OVCAR3 (Figure 3D, E). However, the treatments did not substantially alter the expression of *INK4a* (P16) (Supplementary Figure S8).

INK4 family members exclusively bind to the D-type CDK4 and CDK6 and inhibit their activity, leading to G1 phase cell arrests (Roussel, 1999). It might be that the reduction of *INK4c* gene expression by the treatments reduced G1 arrest. Nevertheless, the subsequent reduction of *PLK1* and increase of *CIP1* gene expression in the cell lines by the treatments might have supported cell cycle arrest during later phases (i.e., S and G2/M).

Apoptosis might be induced because of cell cycle arrest (Pietenpol and Stewart, 2002). Taken together, both aberrant cell cycle and ER stress induced by the niraparib, F7 or niraparib + F7 treatment may have donated to the apoptotic cell death recorded in the OC cell lines under these treatments.

#### 3.4.3 Determining treatment effects on cell migration and F-actin rearrangement

**FIGURE 4:**
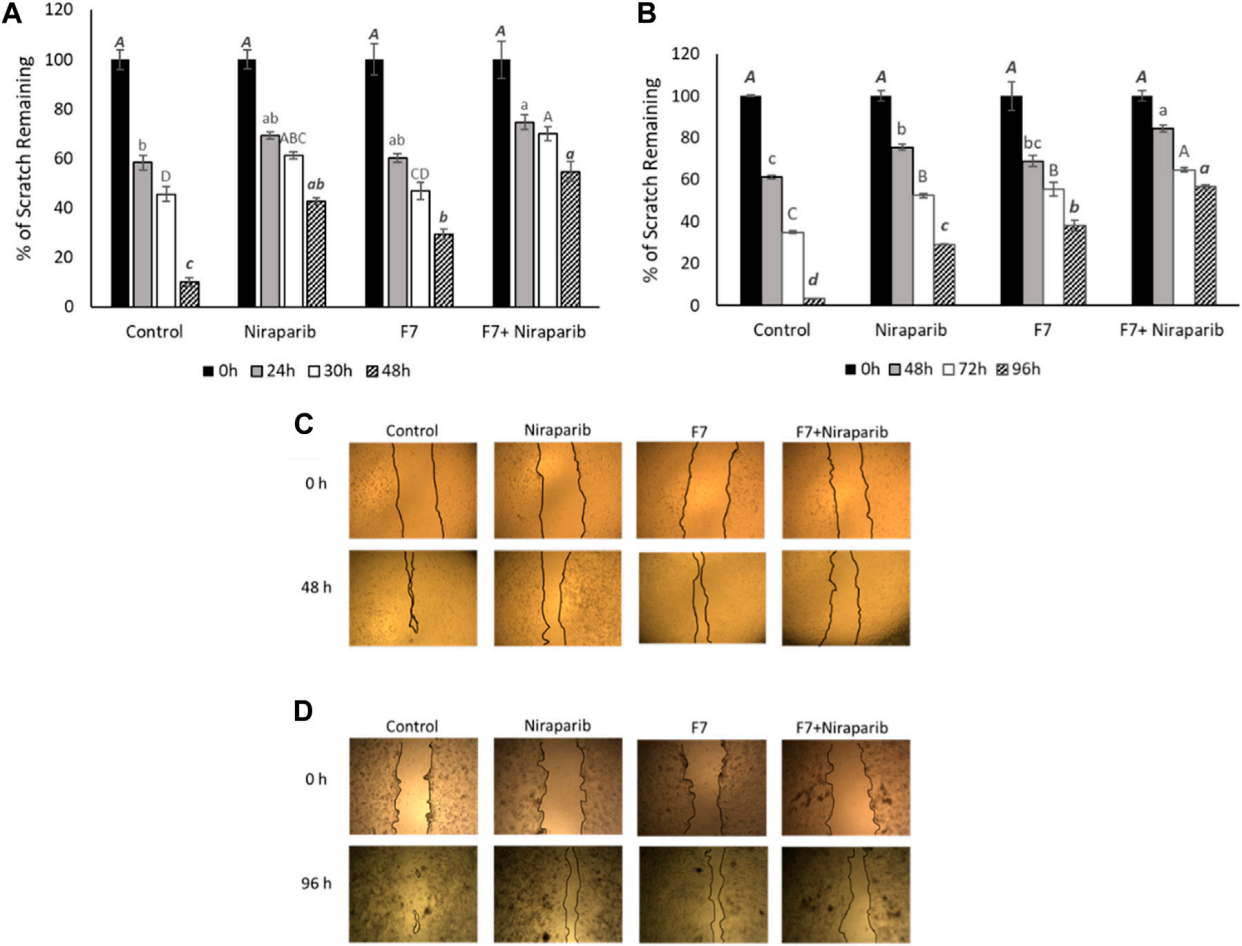
Results of the scratch-wound assay for cell migration, indicating the effects of niraparib (6.1 μg/mL), F7 (11.4 μg/mL) or niraparib + F7 (6.1 μg/mL+11.4 μg/mL, respectively) on Caov3 **(A, C)** and niraparib (14.4 μg/mL), F7 (14.1 μg/mL) or niraparib + F7 (14.4 μg/mL+14.1 μg/mL, respectively) on OVCAR3 **(B, D)** cell lines. Doses of treatments are based on (Shalev et al., 2022). Control is vehicle control (0.35% DMSO+0.5% v/v methanol for Caov3 and 0.7% DMSO+0.7% v/v methanol for OVCAR3). Percent clear scratch area is presented as mean; Error bars indicate ± standard error (*n* = 3) **(A, B)**. One-way ANOVA was performed and means without a common letter denote expression levels that are significantly different by the Tukey-Kramer honest significant difference (HSD; *p* ≤ 0.05). Representative images of the scratch-wound assay in Caov3 **(C)** and OVCAR3 **(D)** cell lines.

The effect of the F7, niraparib or the niraparib + F7 treatments on cell migration was examined at sub-lethal concentrations on Caov3 and OVCAR3 cells using a scratch-wound assay. Almost complete closure of the scratch was obtained in the vehicle control (10.1% of clear area) of Caov3 cells after 48 h (Figures 4A-D). Niraparib inhibited scratch closure, most apparent (42.7% clear area) after 48 h (Figures 4A, C). F7 treatment inhibited scratch closure (29.4% clear area) less than niraparib (Figures 4A, C). However, the combined treatments of niraparib + F7 substantially inhibited cell migration (54.7% clear area), most remarkably at 48 h (Figures 4A, C). In OVCAR3, almost full closure of the scratch was obtained at 96 h in the vehicle control (3.2% clear area; Figures 4B, D). Niraparib or F7 treatments inhibited scratch closure at 96 h (29.2% and 38.0% clear area, respectively; Figures 4B, D) and the combined treatments of niraparib + F7 inhibited cell migration at 96 h to the largest extent (56.6% clear area; Figures 4B, D).

**FIGURE 5:**
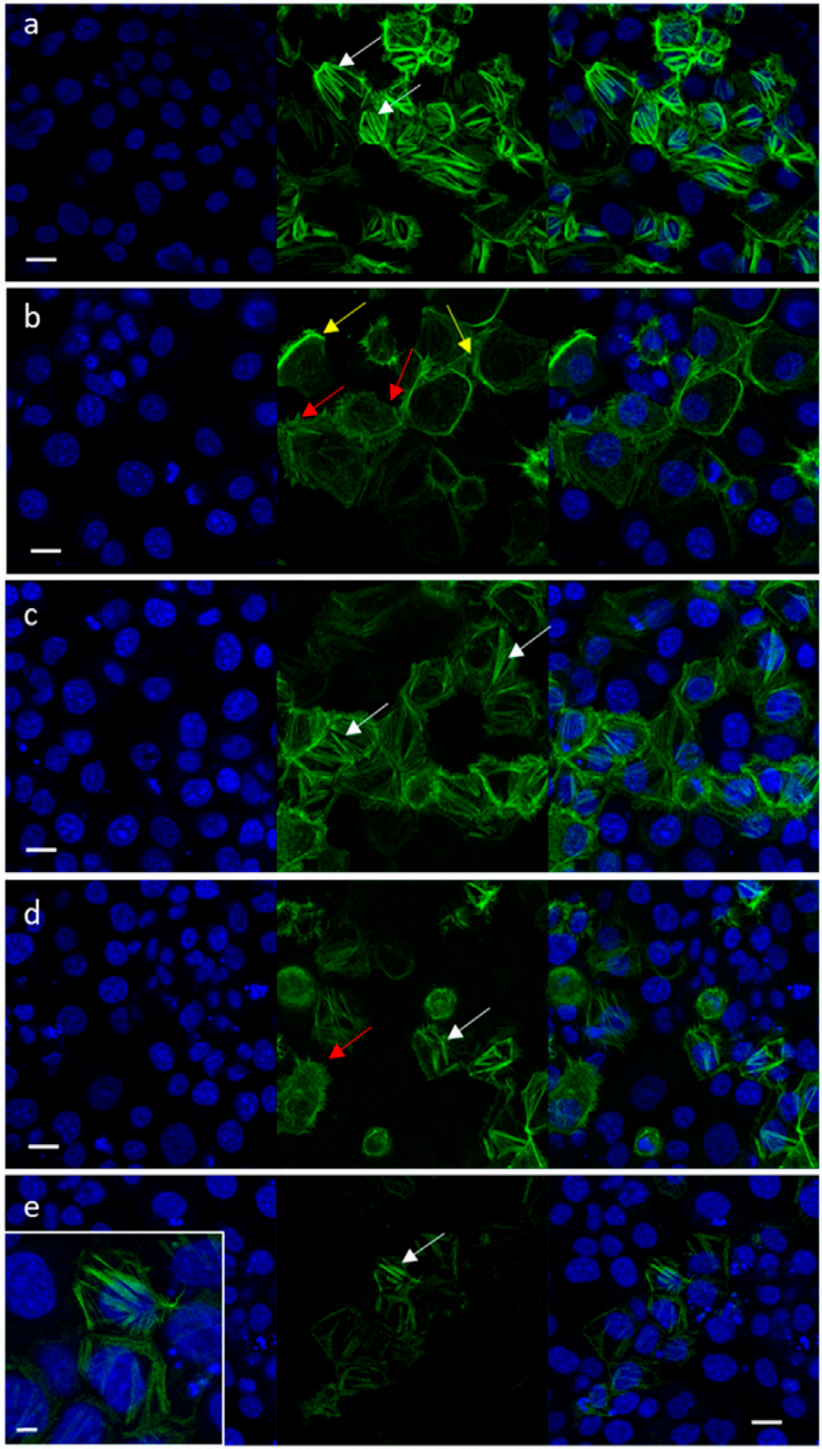
Representative confocal images of OVCAR3 cells non-induced to a mesenchymal phenotype **(A)** and induced to mesenchymal phenotype (RPMI medium contains 5% FBS and 20 ng/mL IL-1β) **(B–E)** following treatment with vehicle control (1.5% v/v methanol; **(B)** niraparib (17.5 μg/mL; **(C)**, F7 (10 μg/mL; **(D)** and niraparib + F7 (7.5 + 6 μg/mL; **(E)** for 16 h. Doses of treatments are based on (Shalev et al., 2022). Cells were labeled with F-ActinGreen 488 and Hoechst (blue). Bars = 20 μm; white arrows point to actin filaments; yellow arrows point to cortical actin filaments; red arrows point to mesenchymal phenotype cells. Inset in **(E)** shows the restored actin filament in higher resolution; bar = 5 µm. In Supplementary Figure S9 are representative images of green signal in cells generated by ImageJ (version 1.53a).

Cell migration is highly affected by F-actin rearrangements (Schaks et al., 2019). Hence, the effects of niraparib, F7 and niraparib + F7 treatments on F-actin cytoskeleton rearrangement was determined after induction for mesenchymal phenotype (García-Morales et al., 2020; Shalev et al., 2022). Previously it was found that only OVCAR3 is substantially responding to the induction to mesenchymal phenotypes (Shalev et al., 2022) and therefore only OVCAR3 was examined for F-actin cytoskeleton rearrangement. In non-induced OVCAR3 cells with epithelial characteristics actin filaments were observed as thick filaments (123 ± 6^a^ pixels) across the cell cytoplasm (Figure 5A; Supplementary Figure S9). In contrast, in OVCAR3 cells induced for mesenchymal phenotypes (Figures 5B–E) in cells treated with the vehicle control, F-actin filaments were fewer (28 ± 9^b^ pixels) and mostly at the cell periphery (Figure 5B; Supplementary Figure S9). Niraparib and F7 treatments after induction led to the rearrangement of the actin-related mesenchymal phenotypes with F-actin filaments evident across the cells (100 ± 12^a^ and 110 ± 11^a^ pixels respectively; Figure 5C, D; Supplementary Figure S9). Yet, with the F7 treatment, few treated cells remained with mesenchymal phenotypes (Figure 5D). However, in the combined niraparib + F7 treatment all stained cells contained thick F-actin elements but to a lesser extent than in the other two treatments or non-induced control (37 ± 6^b^ pixels; Figure 5E; Supplementary Figure S9).

These results, that suggest F-actin is affected by the treatments, are also supported by gene expression results. For example, niraparib, F7 and niraparib + F7 treatments reduced *BMP4* gene expression in OVCAR3 (Supplementary Figure S3). BMP4-related signaling was shown to induce rearrangement of the actin cytoskeleton from the cortical-actin to actin stress fibers (Thériault et al., 2007). In OC, BMP4 is acknowledged as an autocrine ligand and was shown to be associated with induced EMT and increased stemness in OC cells (Fukuda et al., 2020).

#### 3.4.4 Determining treatment effect on the percentage of ALDH + cells in a population

**FIGURE 6:**
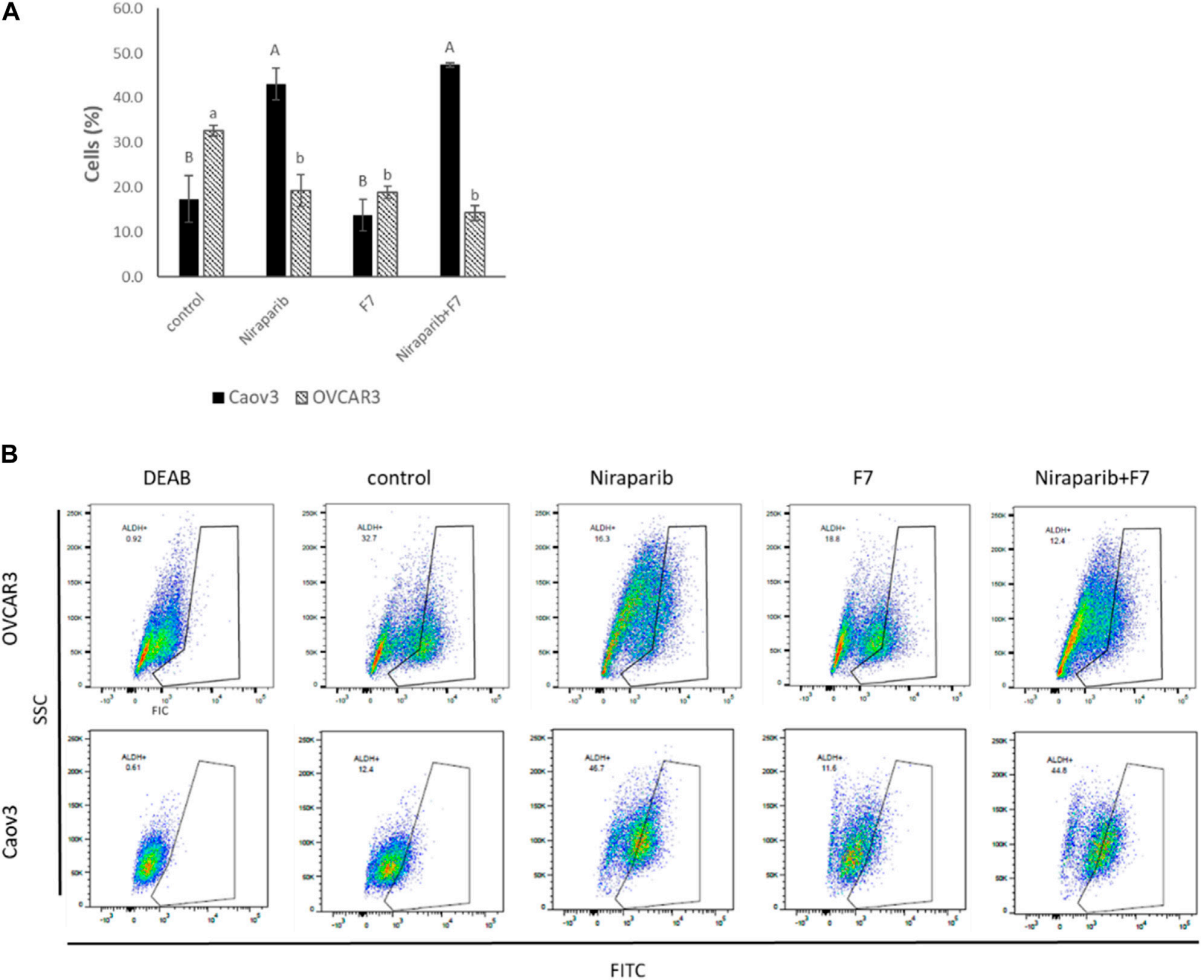
Percentage of ALDH + cells out of total live cells **(A)** in Caov3 and OVCAR3 cell lines following treatments with niraparib (5.1 and 25 μg/mL, respectively), F7 (17 and 24.5 μg/mL, respectively) and niraparib + F7 (5.1 + 17 μg/mL and 25 + 24.5 μg/mL, respectively) for 48 h of incubation. Doses of treatment are based on (Shalev et al., 2022). Control is vehicle control (0.25% DMSO+0.85% methanol v/v for Caov3 and 1.25% DMSO+1.23% methanol v/v for OVCAR3). ALDH activity was determined using Aldefluor assay kit. The treated cells were harvested and analyzed in FACS. Fluorescence channel at 488 nm vs. SSC dot plot was created, while DEAB control was used for gating. Error bars indicate ± standard error (*n* = 3). One-way ANOVA was performed and means without a common letter denote expression levels that are significantly different by the Tukey-Kramer honest significant difference (HSD; *p* ≤ 0.05). Representative flow cytometry dot plots of Caov3 and OVCAR3 cells for FITC **(B)**. Data analysis was preformed using FlowJo software (FlowJo, V 10.8.1, BD Biosciences, CA, United States).

High ALDH activity is closely associated with OC stem-like cells that exhibit enhanced EMT progress and invasiveness responsible for tumor invasion (Li et al., 2018). ALDH activity was examined in Caov3 and OVCAR3 cells treated with IC50 concentrations of niraparib, F7 and niraparib + F7. In Caov3, cells treated with niraparib and niraparib + F7 were enriched with 43.1% and 47.4% ALDH + cells, respectively, while in F7 and the control only 13.7% and 17.4% of the cells were ALDH+, respectively (Figures 6A, B). In OVCAR3, treatments with niraparib, F7 and niraparib + F7 led to reduction in ALDH + cells in comparison to the control, 19.4%, 19.0% and 14.4% versus 32.7% respectively (Figures 6A, B).

Several of the genes described above are associated with the level of ALDH activity. For example, *AJUBA* expression is reduced with the treatments in both cell lines as described above (Supplementary Figure S3). In colon cancer, cell population with lower expression of AJUBA had fewer ALDH + cells (Dommann et al., 2020). In breast cancer cells, higher ALDH activity was found in a FGFR2+ population compared to a FGFR2− population (Kim et al., 2013). Notably, since we found that in Caov3, treatment with F7 does not lead to substantial ALDH + cells enrichment, unlike with the niraparib and niraparib + F7 treatments, it might be that treatment with F7 only might be preferred in this respect over the combined niraparib + F7 treatment. It is possible that F7 alters additional pathways not affected by niraparib or the combined niraparib + F7 treatments to suppress ALDH activity in Caov3 cells. However, in OVCAR3 cell line all treatments (i.e., niraparib, F7 and niraparib + F7) substantially reduced ALDH + cell proportion in population, suggesting that the examined treatments repress aspects related to OC stem-like features.

#### 3.4.5 Determining treatment effects on PARP cleavage

**FIGURE 7:**
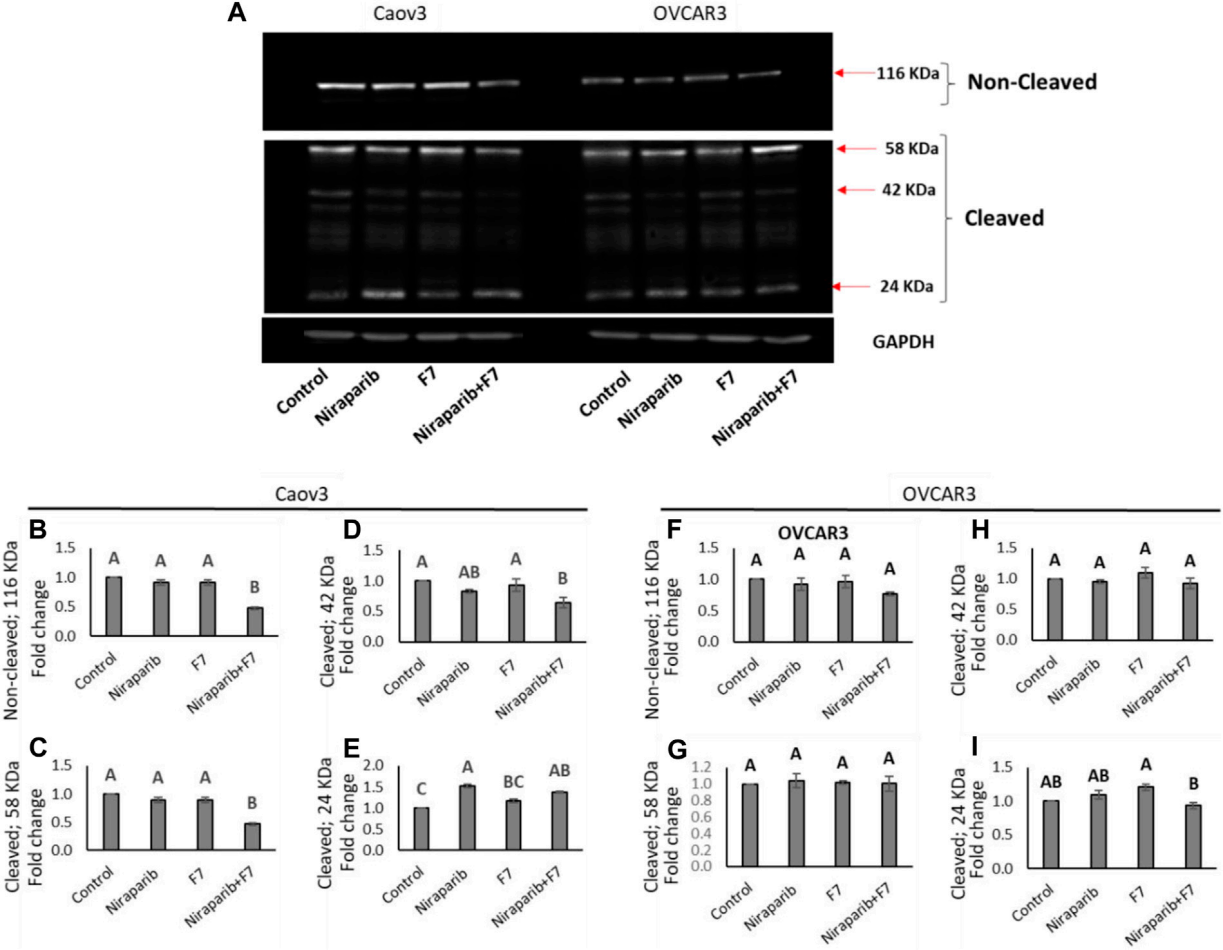
Detection of intact and cleaved PARP1 proteins in Caov3 and OVCAR3 cell lines following niraparib (6 and 25 μg/mL), F7 (17.5 and 20 μg/mL, respectively) and niraparib + F7 (6 + 17.5 μg/mL and 25 + 20 μg/mL, respectively) treatments for 48 h. Doses of treatments are based on (Shalev et al., 2022). **(A)** Representative example of Western blot for PARP1. Intact PARP1 protein and cleaved PARP1 are marked. 1, vehicle control; 2, niraparib; 3, F7; 4, niraparib + F7. GAPDH loading control intensity was similar in all treatments and control. **(B–E)** Quantification of PARP1 non-cleaved and PARP1 cleaved protein bands intensity in the Caov3 cell line **(F–I)** Quantification of PARP1 non-cleaved and PARP1 cleaved protein bands intensity in the OVCAR3 cell line. Spot intensities were quantified using the software ImageJ (version 1.53a). Error bars indicate ± standard error (n = 2 biological replicated and 2 technical replicated). One-way ANOVA was performed and means without a common letter denote expression levels that are significantly different by the Tukey-Kramer honest significant difference (HSD; *p* ≤ 0.05). In Supplementary Figures S11–S13 are pictures of the Western blot gels.

The relative intensity of PARP1 non-cleaved band were significantly reduced in Caov3 cell line with niraparib + F7 treatment (Figures 7A,B). Accordingly, in this treatment, the intensity of the cleaved ∼24 kDa band was significantly increased (Figure 7E) and those of the ∼58 and ∼42 kDa significantly reduced (Figures 7C, D). Significant increase in the ∼24 kDa band was also evident with niraparib treatment, but the levels of the non-cleaved PARP1 band were not significantly affected (Figure 7E). In OVCAR3, however, we could not detect a significant change in the intensity of the non-cleaved and cleaved PARP1 bands with the treatments in comparison to control (Figure 7A; Figures 7F–I).

In line with the increased PARP1 cleavage in Caov3 cells by the niraparib + F7 treatment, this treatment reduced, for example, *PITX2* gene expression in this cell line (Supplementary Figure S5). The involvement of PITX2 was previously demonstrated in PARP cleavage (Zhang et al., 2013). Its cleavage was increased in PITX2 knockdown-human esophageal squamous cell carcinoma cells after irradiation or cisplatin treatments (Zhang et al., 2013).

Notably, in our study, the intensity of the non-cleaved PARP1 was reduced and that of the 24 kD cleaved PARP1-fragment was increased, suggesting a caspase-3 activity signature (Chaitanya et al., 2010). PARP inhibitors bind the catalytic pocket, where they directly interfere with ADP-ribosylation (Spiegel et al., 2021). It might be that in the combined treatment of niraparib + F7, PARP1 is inhibited by both binding to its catalytic domain and its increased cleavage.

## 4 Conclusion

To better characterize the effect of F7 and/or niraparib treatment in OC, we focused on genetic pathways that were significantly and differentially expressed in both cell lines with niraparib + F7 treatment. The expression profile of some of the key genes in these pathways was validated by qPCR and further characterization of the effect of the treatments on cell phenotypes related to these differentially expressed genes was shown by various functional tests. Two subsets of effects were examined: the effect on cell survival/death following treatment and the effect on the mesenchymal phenotype of the cell population following treatment.

The findings presented here suggest that the activity of the niraparib + F7 treatment results from its impact on various signaling pathways and PARP1 inhibition. Notably, genes were examined at their mRNA steady state levels; however, protein expression or activation may differ from gene expression results. Yet, for many of the genes, alterations in mRNA expression levels were already associated with malignant properties [e.g., *BMP4* (Thériault et al., 2007); *ID1* (McAllister et al., 2007), *ID2* (Meng et al., 2009); *AREG* (Tung et al., 2017); *AJUBA* (Dommann et al., 2020); *PITX* (Zhang et al., 2013); *FGFR2* (Cole et al., 2010); *OTX1* (Yu et al., 2014)].

In the present study, for one, apoptotic cell death by the synergistic niraparib + F7 treatment is suggested to be a result of induced ER stress and cell cycle arrest. Moreover, in cells that survive treatment, mesenchymal phenotypes are repressed by the combined treatment, including inhibition of cell migration and changes in the percentage of ALDH + cells in the population. Repression of mesenchymal phenotypes in tumor cells may reduce metastasis (Datta et al., 2021).

Since synergy in many cases is a result of activation of multiple genetic pathways (Chen et al., 2016), co-treatment with niraparib + F7 may promote the robustness of anti-cancer activity of these compounds. Notably, in some cases F7 treatment is the most effective (e.g., on cell cycle arrest, on reduction of proportion of ALDH + cells), in other cases, niraparib treatment is most effective (e.g., on cell migration) and in one case treatment with F7 or niraparib only is clearly superior to the combined treatment (i.e., on F-actin organization). The effectiveness also differs between cell lines (e.g., Caov3 cell cycle arrest with F7+niraparib is dominated by F7, and that of OVCAR3 by niraparib). Accordingly, F7 might alter some signaling pathways and niraparib others. Significantly, combined treatment with niraparib + F7 promotes PARP1 cleavage, whereas niraparib only inhibits PARP1 by binding to the catalytic pocket (Spiegel et al., 2021).

Complex inter-pathway dependencies exist among many pathways; hence, it is also important to consider pathway–pathway interactions in drug synergy (Chen et al., 2016). Indeed, many of the pathways affected by the niraparib + F7 synergy are associated with each other. In the examples described above, Hippo/Wnt, TGF-β/Activin and MAPK signaling pathways are entangled in a pathway–pathway interaction network that induces apoptotic cell death and represses mesenchymal phenotypes. Combinations of PARP1 inhibitors and the F7 cannabis preparation should be further examined for efficacy in animal studies and clinical trials.

## Data Availability

The RNA-Seq data presented in the study are deposited in the NCBI sequence read archive (SRA) as bioproject PRJNA1053662 and biosamples: SAMN38879436, SAMN38879437, SAMN38879438, SAMN38879439, SAMN38879440, SAMN38879441, SAMN38879442, SAMN38879443 for 161-1,161-2,161-3,161-4,75-1,75-2,75-3 and 75-4 respectively.
