# Supplementary material for: Integrated transcriptome and cell phenotype analysis suggest involvement of PARP1 cleavage, Hippo/Wnt, TGF-β and MAPK signaling pathways in ovarian cancer cells response to cannabis and PARP1 inhibitor treatment
Source: Front Genet. 2024 Jan 23;15:1333964. doi: 10.3389/fgene.2024.1333964 (PMC10844430; doi:10.3389/fgene.2024.1333964)
Supplement: Supplementary file 2 [file DataSheet1.docx]

**Supplementary Materials:**

**Integrated Transcriptome and cell phenotype analysis suggest involvement of PARP1 cleavage, Hippo/Wnt, TGF-β and MAPK Signaling Pathways in Ovarian Cancer Cells response to Cannabis and PARP1 Inhibitor treatment**

Nurit Shalev, Michelle Kendall, Navin Kumar, Sudeep Tiwari, Seegehalli M. Anil, Hagit Hauschner,
Savvemala G. Swamy, Adi Faigenboim, Eduard Blausov, Bruce E. Kendall and Hinanit Koltai

**
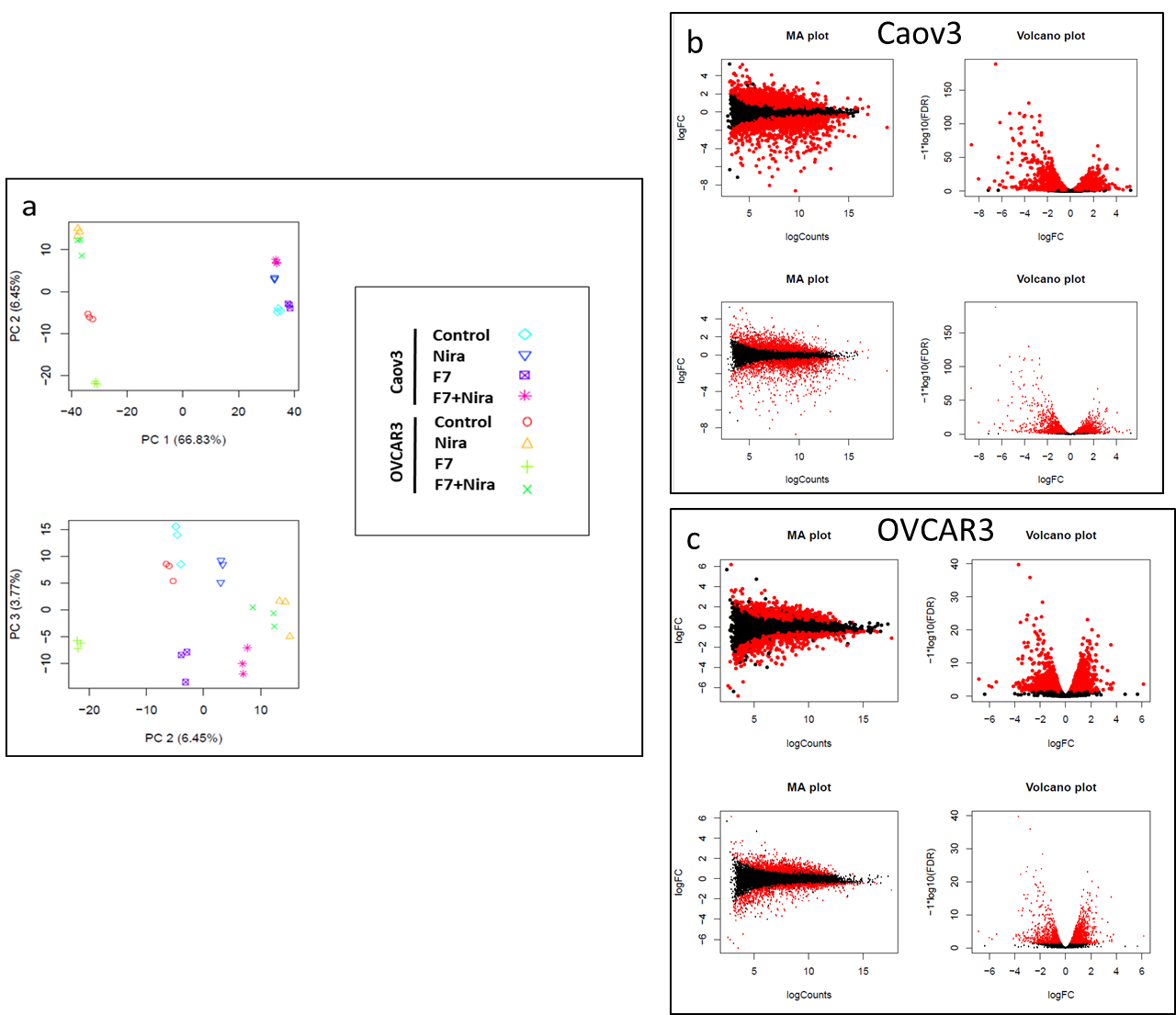
**

**Figure S1.** (a) Principal components analysis (PCA) of all samples based on normalized gene counts (CPM followed by a log2 transform). Caov3 and OVCAR3 cells were treated with niraparib (Nira), F7 or the F7+niraparib combination, in comparison to vehicle control (control). The last digit indicates the replicate number (1-3). The percentages on each axis represent the percentages of variation explained by the principal components. (b) Volcano and MA plots for Caov3 (c) Volcano and MA plots for OVCAR3. Volcano plot: the x-axis represents the log2 fold change and the y-axis represents –log10 (FDR) of each gene. MA plot displaying the log fold-change compared with mean expression counts.


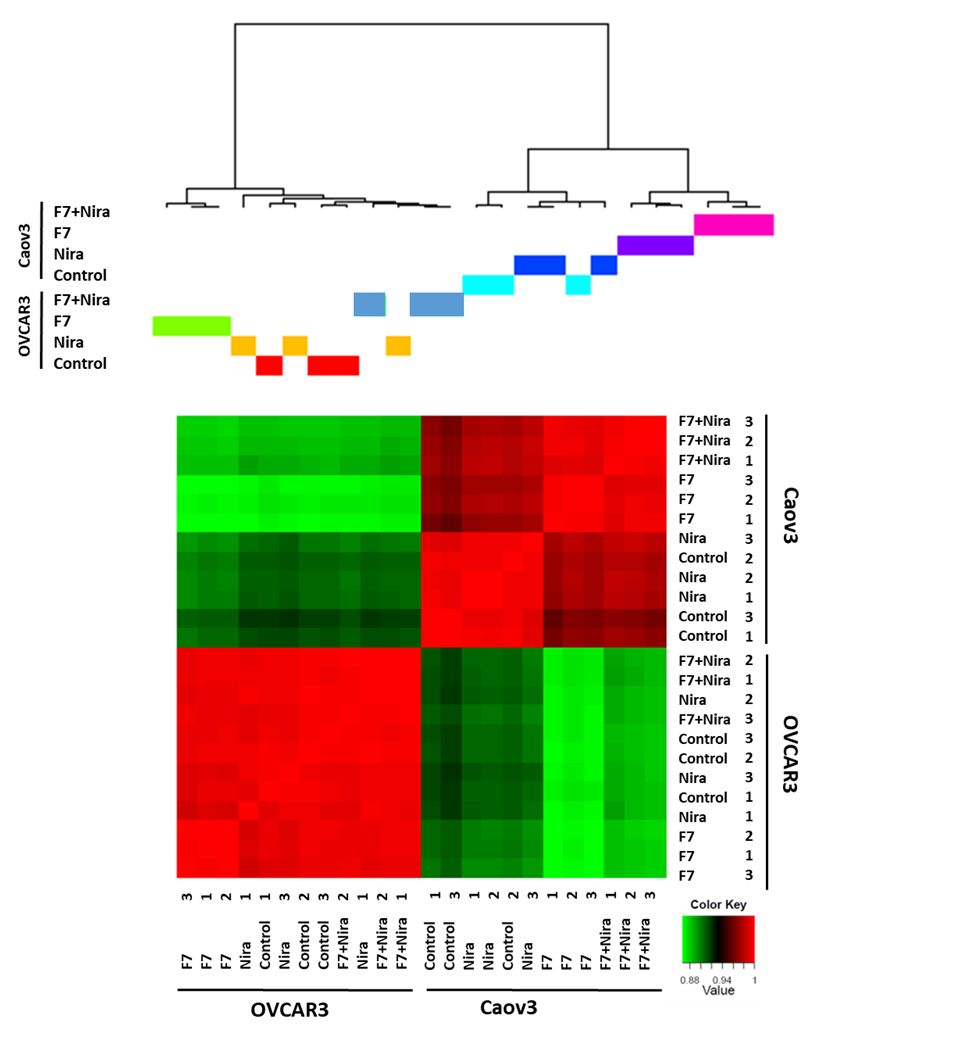


**Figure S2.** Hierarchical clustering of all samples based on the genes significantly and differentially expressed in Caov3 and OVCAR3 cells treated with niraparib (Nira), F7 or the F7+niraparib synergistic combination, in comparison to the vehicle control (control). The last digit indicates the replicate number (1-3). Hierarchical clustering was calculated using Pearson correlations out of dissimilarity tables, among the four conditions based on genes expression (counts per million [CPM]) followed by a log2 transform. Colors indicate the correlation values calculated by Pearson correlations with R software. Correlation ranges from -1 to 1, where -1 indicates anti-correlation (or negative correlation), 0 no correlation and 1 correlation (or positive correlation).


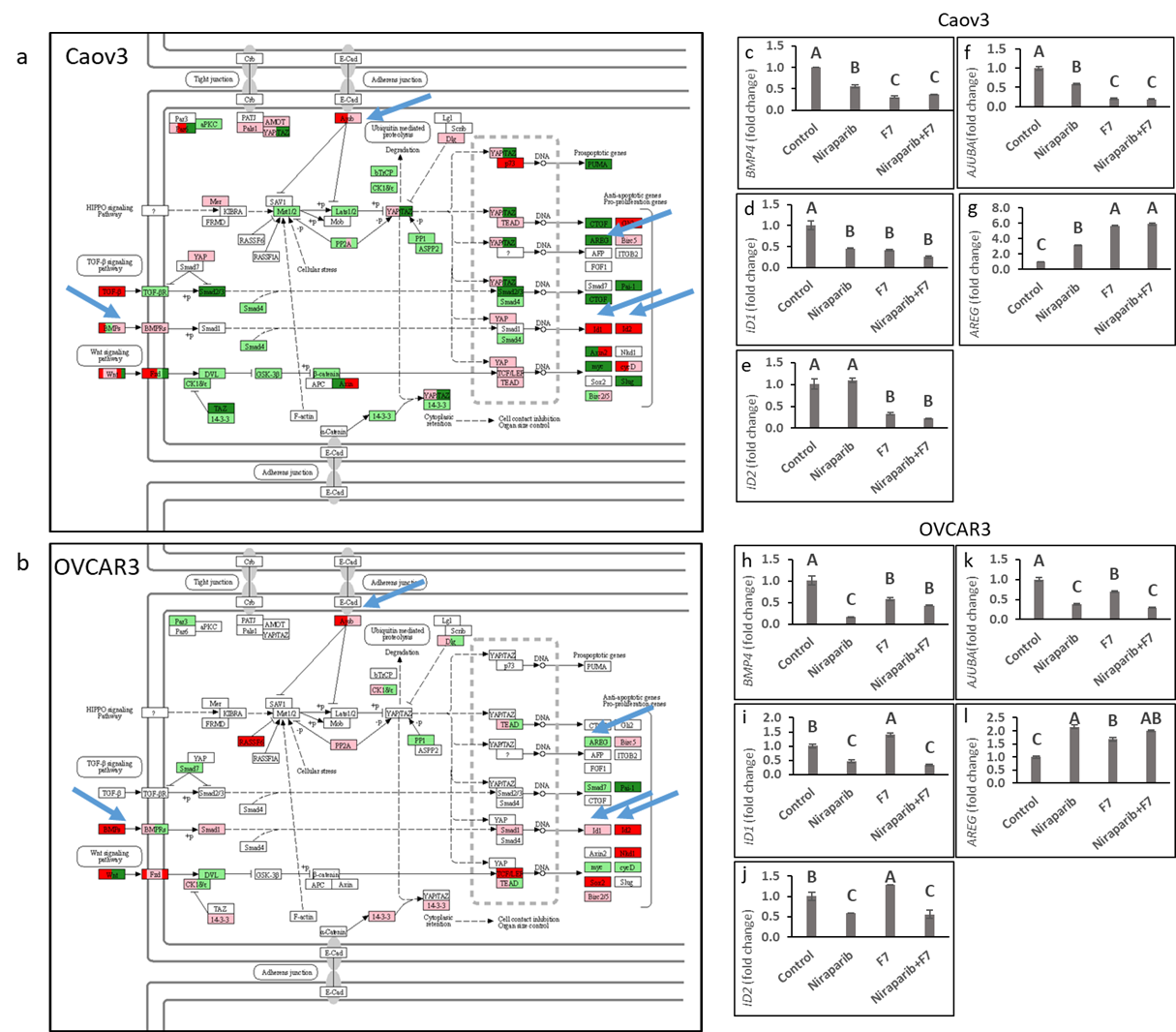


**Figure S3.** Genetic pathways of genes in the Hippo signaling pathway (hsa04390), differentially expressed in Caov3 (a) and OVCAR3 (b) cells treated with F7+niraparib treatment vs. the vehicle control. Pathways determined by KEGG (http://www.genome.jp/kegg/). Green boxes—significantly upregulated genes; red boxes—significantly downregulated genes (DESeq2; more than 2 fold and padj < 0.05). Light green boxes—non-significantly upregulated genes; pink boxes—non-significantly downregulated genes. Boxes of various colors (red, pink, green, light green) denote genes with multiple gene annotations encompassing up and downregulated genes. Blue arrows point to genes further examined by qPCR and functional tests. mRNA steady state level based on quantitative PCR in Caov3 (c-g) and OVCAR3 (h-l) cell lines treated for 6 h with niraparib (6 or 25 µg/mL for Caov3 or OVCAR3, respectively), F7 (17.5 or 20 µg/mL for Caov3 or OVCAR3, respectively), or a combination of niraparib+F7 at the corresponding concentrations, relative to control. Quantitative PCR was used to determine gene transcript values as a difference between the target genes and a reference gene (HPRT) using the 2^ΔΔCt^ method. . Control is the vehicle control (0.3% DMSO+0.87% v/v methanol for Caov3 and 1.25% DMSO+1% v/v methanol for OVCAR3). Error bars indicate ± standard error. (n = 3). One-way ANOVA was performed and means without a common letter denote expression levels that are significantly different by the Tukey-Kramer honest significant difference (HSD; P ≤ 0.05).


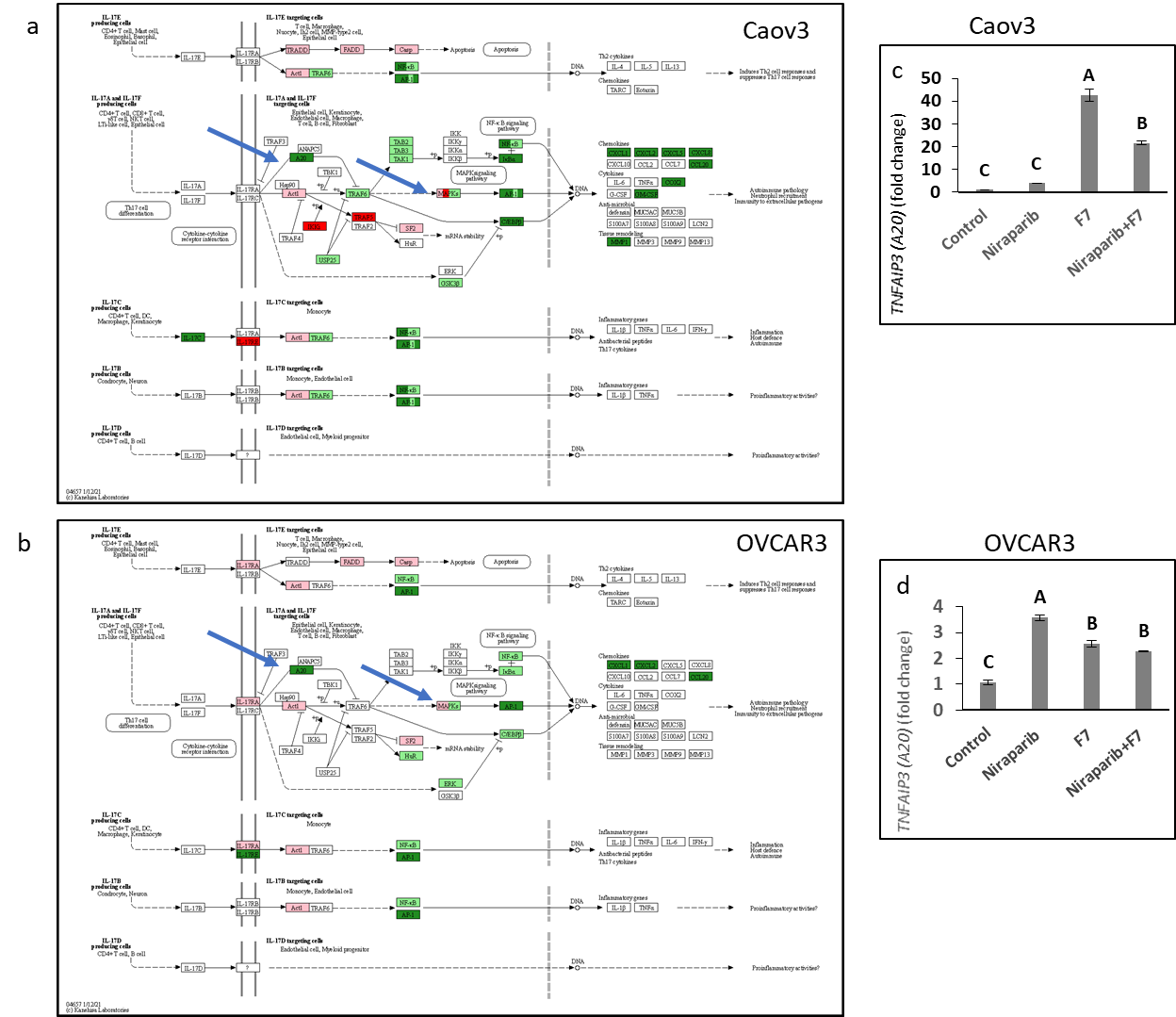


**Figure S4.** Genetic pathways of genes in the IL17 signaling pathway (hsa04657), differentially expressed in Caov3 (a) and OVCAR3 (b) cells treated with F7+niraparib treatment vs. vehicle control. Pathways were determined with KEGG (http://www.genome.jp/kegg/). Green boxes—significantly upregulated genes; red boxes—significantly downregulated genes (DESeq2; more than 2 fold and padj < 0.05). Light green boxes—non-significantly upregulated genes; pink boxes—non-significantly downregulated genes. Boxes of various colors (red, pink, green, light green) denote genes with multiple gene annotations encompassing up and downregulated genes. Blue arrows point to genes further examined by qPCR and functional tests. mRNA steady state level based on quantitative PCR in Caov3 (c) and OVCAR3 (d) cell lines treated for 6 h with niraparib (6 or 25 µg/mL for Caov3 or OVCAR3, respectively), F7 (17.5 or 20 µg/mL for Caov3 or OVCAR3, respectively), or a combination of niraparib+F7 at the corresponding concentrations, relative to control. Quantitative PCR was used to determine gene transcript values as a difference between the target genes and a reference gene (HPRT) using the 2^ΔΔCt^ method. . Control is the vehicle control (0.3% DMSO+0.87% v/v methanol for Caov3 and 1.25% DMSO+1% v/v methanol for OVCAR3). Error bars indicate ± standard error. (n = 3). One-way ANOVA was performed and means without a common letter denote expression levels that are significantly different by the Tukey-Kramer honest significant difference (HSD; P ≤ 0.05).


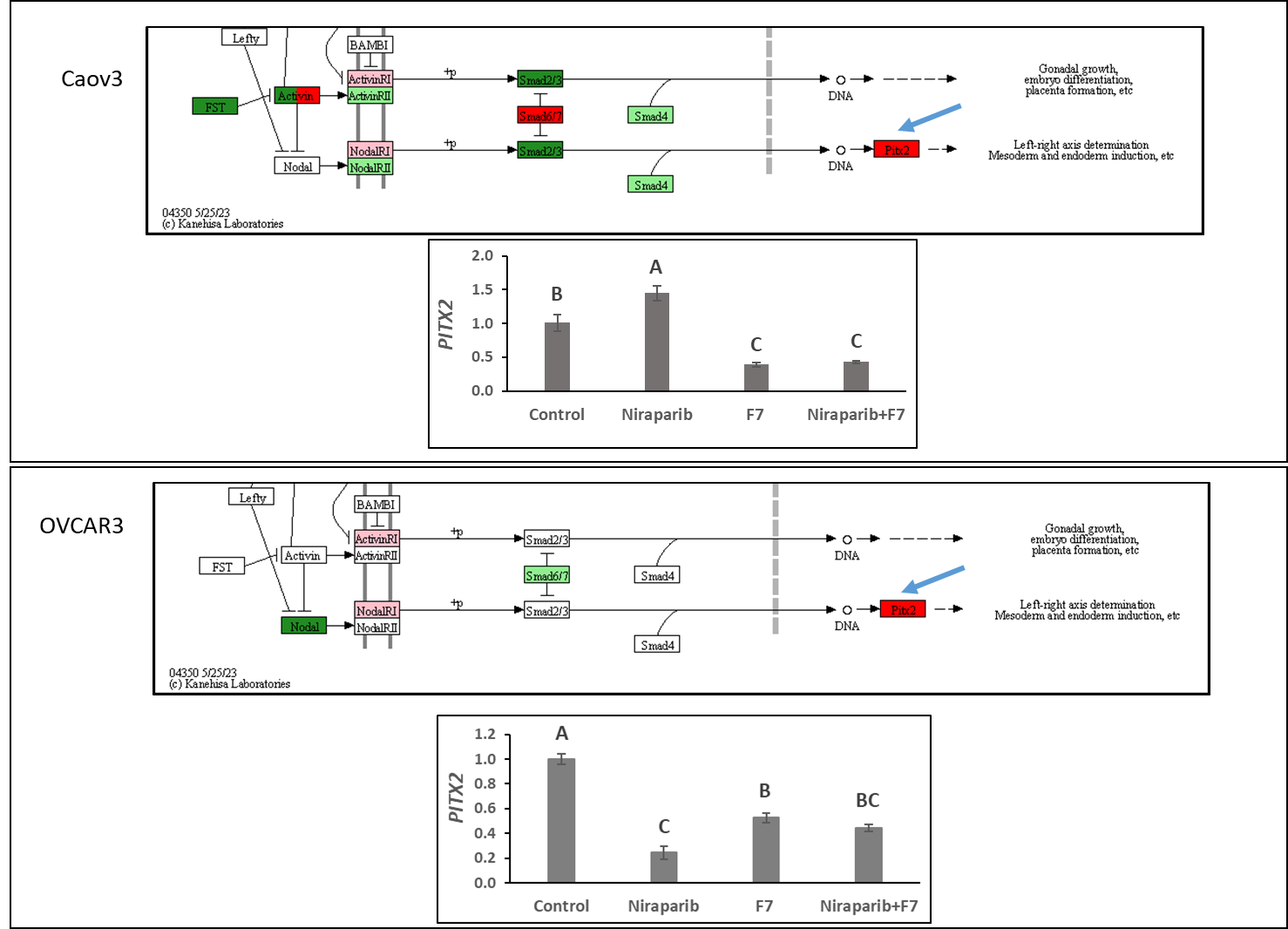


d

c

b

a

**Figure S5.** Genetic pathways of genes in part of the TGF-beta signaling pathway (hsa04350) differentially expressed in Caov3 (a) and OVCAR3 (b) cells treated with F7+niraparib treatment vs. the vehicle control. Pathways were determined with KEGG (http://www.genome.jp/kegg/). Green boxes—significantly upregulated genes; red boxes—significantly downregulated genes (DESeq2; more than 2 fold and padj < 0.05). Light green boxes—non-significantly upregulated genes; pink boxes—non-significantly downregulated genes. Boxes of various colors (red, pink, green, light green) denote genes with multiple annotations encompassing up and downregulated genes. Blue arrows point to genes further examined by qPCR and functional tests. mRNA steady state level based on quantitative PCR in Caov3 (c) and OVCAR3 (d) cell lines treated for 6 h with niraparib (6 or 25 µg/mL for Caov3 or OVCAR3, respectively), F7 (17.5 or 20 µg/mL for Caov3 or OVCAR3, respectively), or a combination of niraparib+F7 at the corresponding concentrations, relative to control. Quantitative PCR was used to determine gene transcript values as a difference between the target genes and a reference gene (HPRT) using the 2^ΔΔCt^ method. . Control is the vehicle control (0.3% DMSO+0.87% v/v methanol for Caov3 and 1.25% DMSO+1% v/v methanol for OVCAR3). Error bars indicate ± standard error. (n = 3). One-way ANOVA was performed and means without a common letter denote expression levels that are significantly different by the Tukey-Kramer honest significant difference (HSD; P ≤ 0.05).


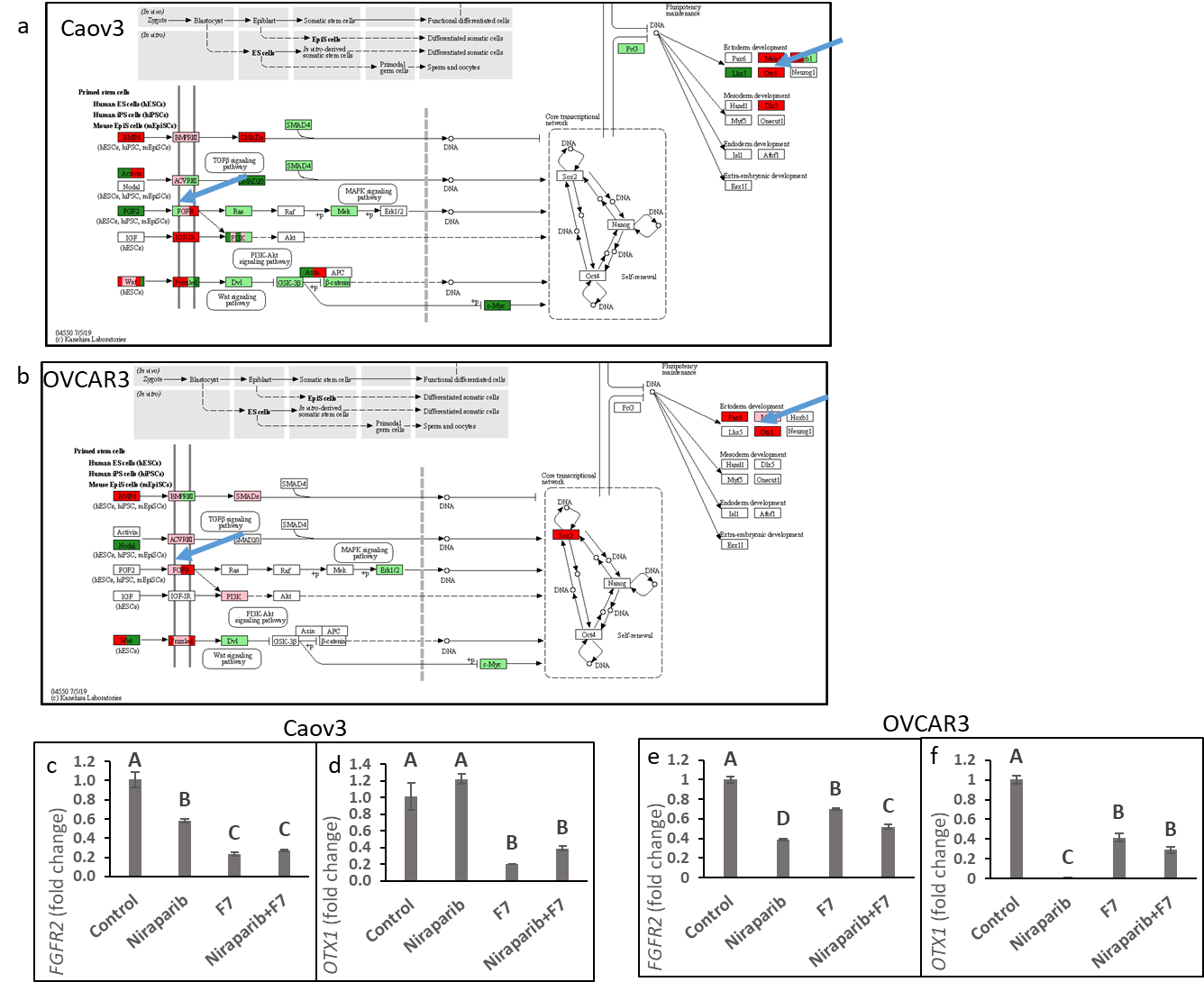


**Figure S6.** Genetic pathways of genes in part of the Signaling pathways regulating pluripotency of stem cells (hsa04550) differentially expressed in Caov3 (a) and OVCAR3(b) cells treated with F7+niraparib treatment vs. vehicle control. Pathways were determined with KEGG (http://www.genome.jp/kegg/). Green boxes—significantly upregulated genes; red boxes—significantly downregulated genes (DESeq2; more than 2 fold and padj < 0.05). Light green boxes—non-significantly upregulated genes; pink boxes—non-significantly downregulated genes. Boxes of various colors (red, pink, green, light green) denote genes with multiple gene annotations encompassing up and downregulated genes. Blue arrows point to genes further examined by qPCR and to functional tests. mRNA steady state level based on quantitative PCR in Caov3 (c,d) and OVCAR3 (e,f) cell lines treated for 6 h with niraparib (6 or 25 µg/mL for Caov3 or OVCAR3, respectively), F7 (17.5 or 20 µg/mL for Caov3 or OVCAR3, respectively), or a combination of niraparib+F7 at the corresponding concentrations, relative to control. Quantitative PCR was used to determine gene transcript values as a difference between the target genes and a reference gene (HPRT) using the 2^ΔΔCt^ method. . Control is the vehicle control (0.3% DMSO+0.87% v/v methanol for Caov3 and 1.25% DMSO+1% v/v methanol for OVCAR3). Error bars indicate ± standard error. (n = 3). One-way ANOVA was performed and means without a common letter denote expression levels that are significantly different by the Tukey-Kramer honest significant difference (HSD; P ≤ 0.05).


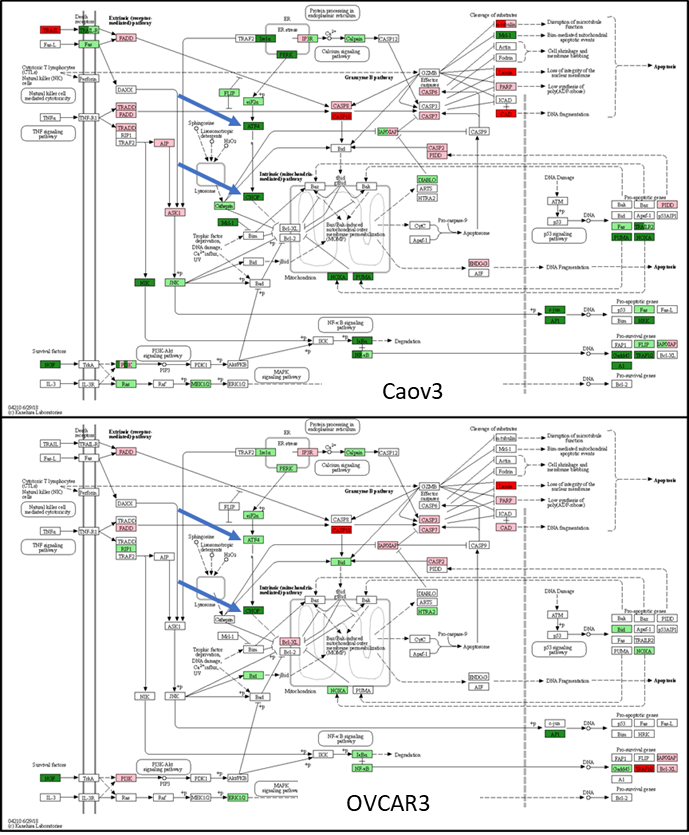


**Figure S7.** Genetic pathways of genes in the apoptosis pathway (hsa04210), differentially expressed in Caov3 and OVCAR3 cells treated with F7+niraparib treatment vs. the vehicle control. Pathways were determined with KEGG (http://www.genome.jp/kegg/). Green boxes—significantly upregulated genes; red boxes—significantly downregulated genes (DESeq2; more than 2 fold and padj < 0.05). Light green boxes—non-significantly upregulated genes; pink boxes—non-significantly downregulated genes. Boxes with various colors (red, pink, green, light green) denote genes with multiple gene annotations encompassing upregulated and downregulated genes; light yellow boxes denote genes with multiple gene annotations encompassing both non-significant upregulated and non-significant downregulated genes. Blue arrows point to genes further examined by qPCR and functional tests.


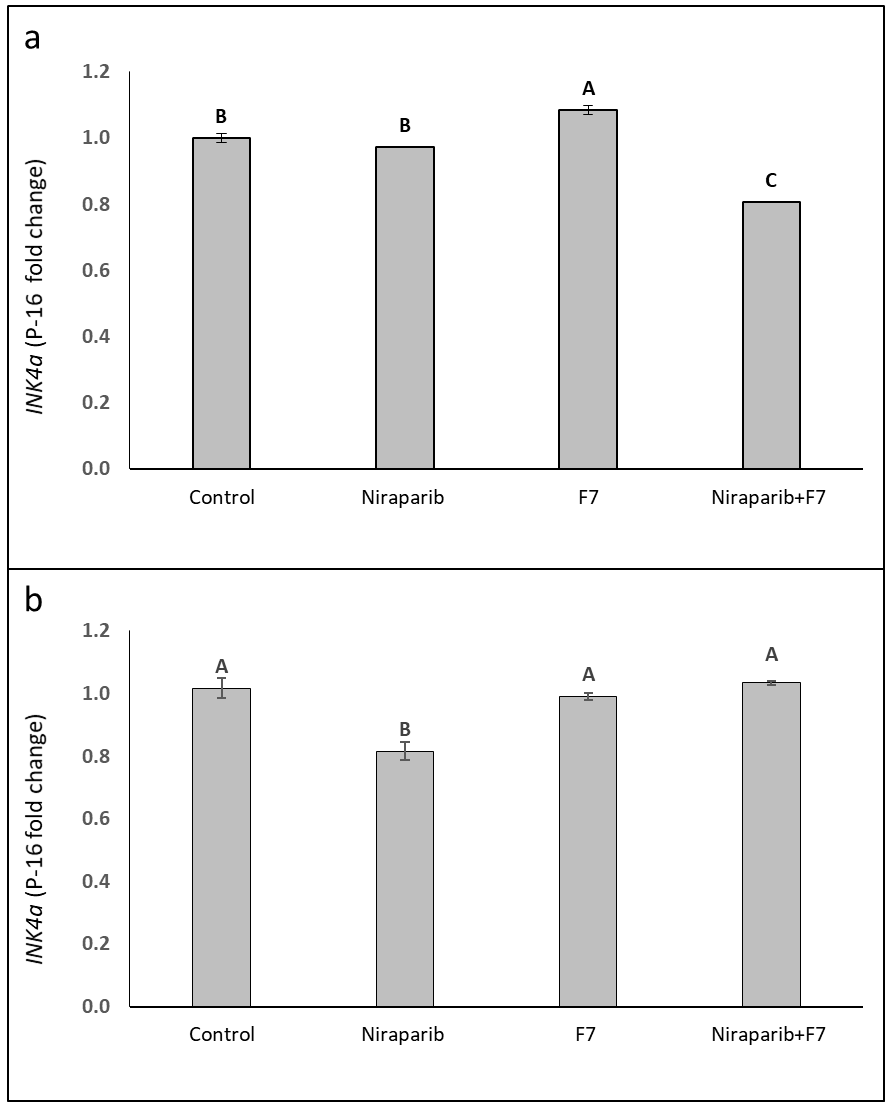


**Figure S8.** Determination of the *INK4a* (P16) RNA steady state level based on quantitative PCR in Caov3 (a) and OVCAR3 (b) cell lines at 6 h of treatment with niraparib (6 or 25 µg/mL for Caov3 or OVCAR3, respectively), F7 (17.5 or 20 µg/mL for Caov3 or OVCAR3, respectively), or a combination of niraparib+F7 (in the above designated concentrations), relative to control. Gene transcript values were determined by quantitative PCR as a difference between the target gene versus a reference gene (HPRT). Values were calculated relative to the average expression of target genes in treated versus control using the 2-ΔΔCt method. Control is the vehicle control (1.00% v/v methanol + 0.60% v/v DMSO). (n = 3). In Supplementary Table S7, different letters stand for expression levels that are significantly different from all combinations of pairs by Tukey-Kramer honest significant difference (HSD; P ≤ 0.05).

**
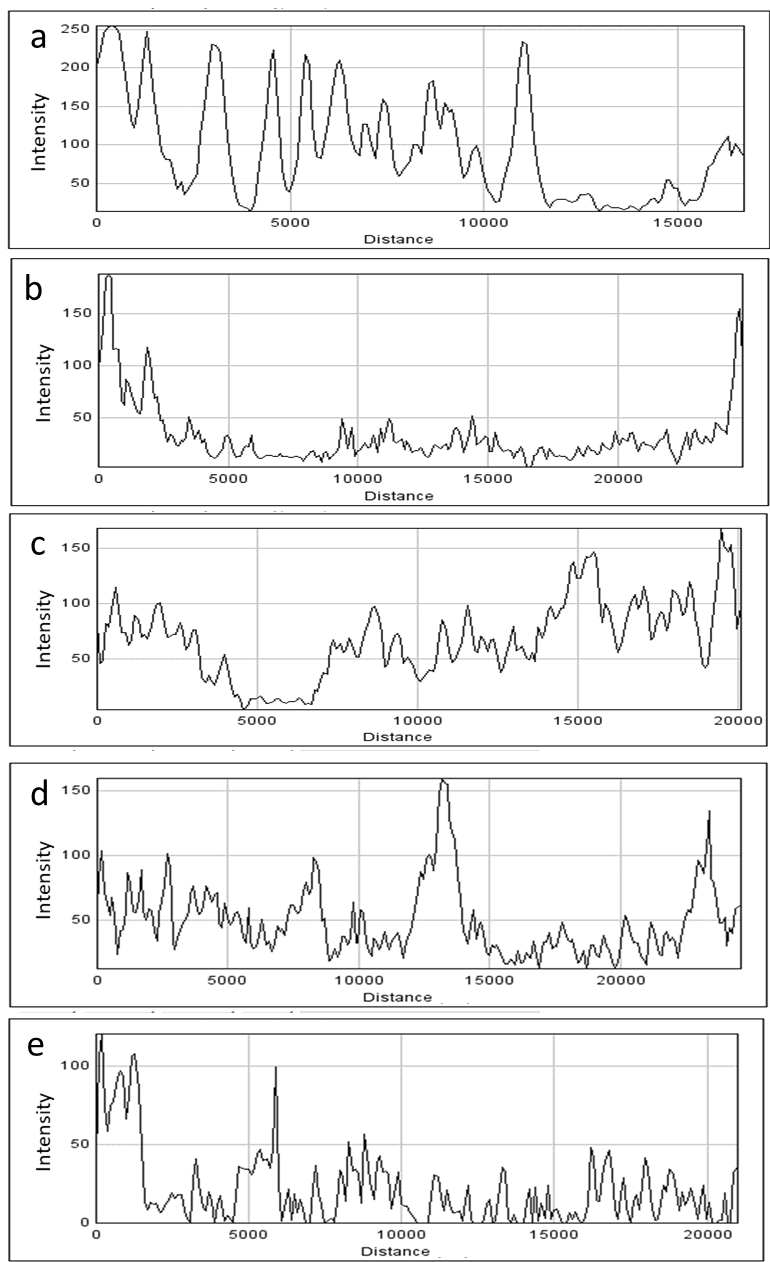
**

**Figure S9.** Representative images of cellular green signal generated by ImageJ (version 1.53a) of OVCAR3 cells non-induced to a mesenchymal phenotype (a) and induced to mesenchymal phenotype (RPMI medium contains 5% FBS and 20ng/mL IL-1β) (b-e) following treatment with vehicle control (1.5% v/v methanol; b) niraparib (17.5 µg/mL; c), F7 (10 µg/mL; d) and niraparib+F7 (7.5 + 6 µg/mL; e) for 16 h. Y axis is intensity of the green signal. X axis is the measured distance in pixels. Representative confocal images are in Figure 5.


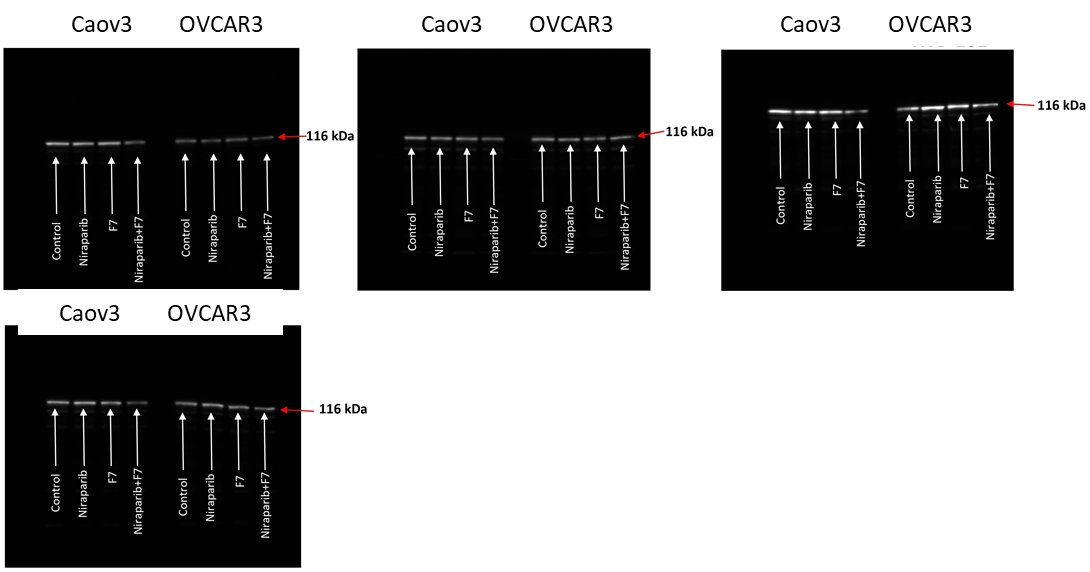


**Figure S11.** Western blotting of PARP 1 for non-cleaved protein in Caov3 and OVCAR3 cell lines.


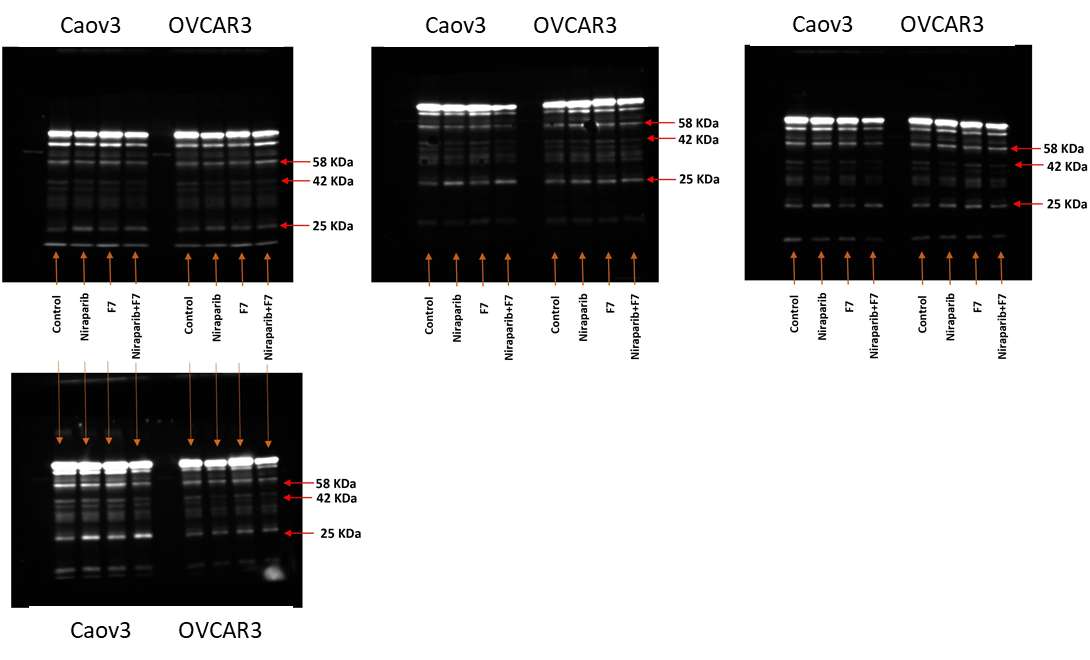


**Figure S12.** Western blotting of PARP1 for non-cleaved and cleaved proteins in Caov3 and OVCAR3 cell lines.


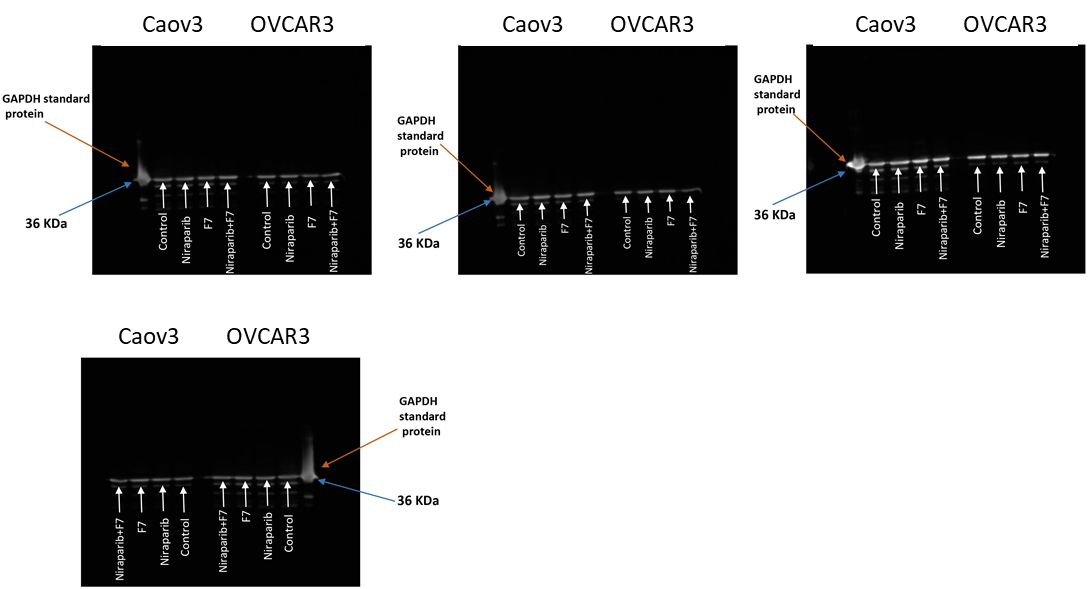


**Figure S13.** Western blotting of GAPDH protein in Caov3 and OVCAR3 cell lines.

**Table S1.** List of gene primers used for quantitative PCR analysis. **.**

| **Gene** | **Gene ID** | **Primers (F-forward, R-reverse)** | |
| --- | --- | --- | --- |
| AJUBA | 84962 | F- TTTGTTTGCTGCTCTTGTGG | R- CTTCCCCATTGCTTGTAGGA |
| AREG | 374 | F- CCACAGTGCTGATGGATTTG | R- AGCCAGGTATTTGTGGTTCG |
| BMP4 | 652 | F- GGAGGAGGAGGAAGAGCAGA | R- CACTGGTCCCTGGGATGTTC |
| FGFR2 | 2263 | F- CTTCACAGCCACTTTGGTCA | R- AAGCTGCTGAAGGAAGGACA |
| ID1 | 3397 | F- AAACGTGCTGCTCTACGACA | R- GGGGGTTCCAACTTCGGATT |
| ID2 | 3398 | F- CCCACTATTGTCAGCCTGCA | R- CCACACAGTGCTTTGCTGTC |
| OTX1 | 5013 | F- CTTAAGCCTCCCCTTCCAGT | R- CACGCCAGTTAGTGACTCCA |
| PITX2 | 5308 | F- CCCTACGACGACATGTACCC | R- GTGGGGAAAACATGCTCTGT |
| TNFAIP3 | 7128 | F- GCTGAAAACGAACGGTGA | R- CATCATTCCAGTTCCGAGT |
| DDIT3 | 1649 | F- AGCAGAGGTCACAAGCACCT | R- CTGGGGAATGACCACTCTGT |
| ATF4 | 468 | F- GCCAAGCACTTCAAACCTCA | R- CTTGCTGTTGTTGGAGGGAC |
| TRIB3 | 57761 | F- CCAAACCTTCAGTGCCTTCC | R- CACCTGATAAGCACCCAAGC |
| CIP1 | 1026 | F- TATGGGGCTGGGAGTAGTTG | R- AGCCGAGAGAAAACAGTCCA |
| PLK1 | 5347 | F- GACAAGTACGGCCTTGGGTA | R- GTGCCGTCACGCTCTATGTA |
| INK4C | 1031 | F- GGACCCAGGACTATCCCTTC | R- TTTAGGGTCCCTTGTTCACG |

**Table S2.** Biological processes enriched with annotated genes (ratio of no. genes upregulated/ no. of genes in the pathway >0.2; P≤0.05) that were upregulated by F7+niraparib treatment vs. the vehicle control and common to both Caov3 and OVCAR3 cell lines.

| **Biological process** | **Gene  Ontology ID** | **No. genes  upregulated** | **No. of genes in the pathway** | **P-Value** | **Corrected P-Value** | **Enrichment** |
| --- | --- | --- | --- | --- | --- | --- |
| protein tyrosine/threonine phosphatase  activity | GO:0008330 | 3 | 10 | 2.94E-06 | 0.000141 | 0.3 |
| PERK-mediated unfolded protein response | GO:0036499 | 3 | 12 | 4.67E-06 | 0.000209 | 0.25 |
| response to gravity | GO:0009629 | 2 | 8 | 0.000215 | 0.003861 | 0.25 |
| response to corticosterone | GO:0051412 | 4 | 17 | 1.30E-07 | 9.15E-06 | 0.235294 |
| MAP kinase tyrosine/serine/threonine  phosphatase activity | GO:0017017 | 3 | 13 | 5.73E-06 | 0.000246 | 0.230769 |
| cellular response to arsenic-containing substance | GO:0071243 | 2 | 9 | 0.000263 | 0.004577 | 0.222222 |

**Table S3.** Biological processes enriched (ratio of no. genes downregulated/ no. of genes in the pathway >0.2; P≤0.05) with annotated genes that were downregulated by the F7+niraparib treatment vs. the control and common to both Caov3 and OVCAR3 cell lines.

| **Biological process** | **Gene  Ontology ID** | **No. genes  downregulated** | **No. of genes in the pathway** | **P-Value** | **Corrected P-Value** | **Enrichment** |
| --- | --- | --- | --- | --- | --- | --- |
| muscular septum morphogenesis | GO:0003150 | 3 | 6 | 7.96E-06 | 0.000622 | 0.5 |
| telencephalon regionalization | GO:0021978 | 3 | 6 | 7.96E-06 | 0.000622 | 0.5 |
| membranous septum morphogenesis | GO:0003149 | 4 | 10 | 4.24E-07 | 5.87E-05 | 0.4 |
| negative regulation of protein localization to microtubule | GO:1902817 | 2 | 5 | 0.000438 | 0.01507 | 0.4 |
| regulation of smooth muscle cell  differentiation | GO:0051150 | 2 | 5 | 0.000438 | 0.01507 | 0.4 |
| retinal blood vessel morphogenesis | GO:0061304 | 2 | 6 | 0.000583 | 0.018211 | 0.333333 |
| prostate epithelial cord arborization  involved in prostate glandular acinus morphogenesis | GO:0060527 | 2 | 6 | 0.000583 | 0.018211 | 0.333333 |
| dorsal/ventral neural tube patterning | GO:0021904 | 3 | 10 | 2.67E-05 | 0.001642 | 0.3 |
| bud elongation involved in lung branching | GO:0060449 | 2 | 7 | 0.000747 | 0.02006 | 0.285714 |
| generation of neurons | GO:0048699 | 2 | 7 | 0.000747 | 0.02006 | 0.285714 |
| positive regulation of phospholipase  activity | GO:0010518 | 2 | 7 | 0.000747 | 0.02006 | 0.285714 |
| negative regulation of norepinephrine secretion | GO:0010700 | 2 | 8 | 0.000931 | 0.022856 | 0.25 |
| myoblast migration | GO:0051451 | 2 | 9 | 0.001134 | 0.024678 | 0.222222 |
| positive regulation of potassium ion transport | GO:0043268 | 2 | 9 | 0.001134 | 0.024678 | 0.222222 |
| regulation of neural precursor cell  proliferation | GO:2000177 | 2 | 9 | 0.001134 | 0.024678 | 0.222222 |
| G protein-coupled adenosine receptor signaling pathway | GO:0001973 | 2 | 9 | 0.001134 | 0.024678 | 0.222222 |
| lung-associated mesenchyme  development | GO:0060484 | 2 | 9 | 0.001134 | 0.024678 | 0.222222 |

**Table S4.** Biological processes enriched (ratio of no. genes upregulated/ no. of genes in the pathway >0.2; P≤0.05) with annotated genes that were upregulated specifically by the F7+niraparib treatment in Caov3.

| **Biological process** | **Gene  Ontology ID** | **No. genes  upregulated** | **No. of genes in the pathway** | **P-Value** | **Corrected P-Value** | **Enrichment** |
| --- | --- | --- | --- | --- | --- | --- |
| positive regulation of action potential | GO:0045760 | 2 | 5 | 0.000969 | 0.032691 | 0.4 |
| neurofibrillary tangle | GO:0097418 | 2 | 5 | 0.000969 | 0.032691 | 0.4 |
| ciliary neurotrophic factor-mediated  signaling pathway | GO:0070120 | 2 | 5 | 0.000969 | 0.032691 | 0.4 |
| RNA 3'-end processing | GO:0031123 | 2 | 5 | 0.000969 | 0.032691 | 0.4 |
| negative regulation of calcium  ion-dependent exocytosis | GO:0045955 | 3 | 9 | 6.78E-05 | 0.004827 | 0.333333 |
| oncostatin-M receptor activity | GO:0004924 | 2 | 6 | 0.001286 | 0.035331 | 0.333333 |
| ADP-ribose diphosphatase activity | GO:0047631 | 2 | 6 | 0.001286 | 0.035331 | 0.333333 |
| leukemia inhibitory factor receptor (LIFR) activity | GO:0004923 | 2 | 6 | 0.001286 | 0.035331 | 0.333333 |
| neurofilament cytoskeleton organization | GO:0060052 | 2 | 7 | 0.001646 | 0.040068 | 0.285714 |
| oncostatin-M-mediated signaling pathway | GO:0038165 | 2 | 7 | 0.001646 | 0.040068 | 0.285714 |
| Notch signaling involved in heart  development | GO:0061314 | 3 | 11 | 0.000111 | 0.006759 | 0.272727 |
| dosage compensation by inactivation of X chromosome | GO:0009048 | 3 | 11 | 0.000111 | 0.006759 | 0.272727 |
| positive regulation of cardiac muscle  contraction | GO:0060452 | 2 | 8 | 0.002048 | 0.044761 | 0.25 |
| dorsal aorta morphogenesis | GO:0035912 | 2 | 8 | 0.002048 | 0.044761 | 0.25 |
| nucleotide-binding oligomerization  domain containing 2 signaling pathway | GO:0070431 | 2 | 8 | 0.002048 | 0.044761 | 0.25 |
| positive regulation of collateral sprouting | GO:0048672 | 2 | 8 | 0.002048 | 0.044761 | 0.25 |
| ciliary neurotrophic factor receptor  binding | GO:0005127 | 2 | 9 | 0.002492 | 0.046983 | 0.222222 |
| positive regulation of triglyceride catabolic process | GO:0010898 | 2 | 9 | 0.002492 | 0.046983 | 0.222222 |
| cellular response to muramyl dipeptide | GO:0071225 | 2 | 9 | 0.002492 | 0.046983 | 0.222222 |
| regulation of neural precursor cell  proliferation | GO:2000177 | 2 | 9 | 0.002492 | 0.046983 | 0.222222 |
| cardiac ventricle morphogenesis | GO:0003208 | 2 | 9 | 0.002492 | 0.046983 | 0.222222 |
| keratinocyte development | GO:0003334 | 2 | 9 | 0.002492 | 0.046983 | 0.222222 |

**Table S5.** Biological processes enriched (ratio of no. genes downregulated/ no. of genes in the pathway >0.2; P≤0.05) with annotated genes that were downregulated specifically by the F7+niraparib treatment in Caov3.

| **Biological process** | **Gene  Ontology ID** | **No. genes  downregulated** | **No. of genes in the pathway** | **P-Value** | **Corrected P-Value** | **Enrichment** |
| --- | --- | --- | --- | --- | --- | --- |
| muscular septum morphogenesis | GO:0003150 | 4 | 6 | 3.66E-06 | 0.000372 | 0.666667 |
| regulation of branching involved in  prostate gland morphogenesis | GO:0060687 | 3 | 6 | 0.000126 | 0.006729 | 0.5 |
| demethylase activity | GO:0032451 | 3 | 6 | 0.000126 | 0.006729 | 0.5 |
| generation of neurons | GO:0048699 | 3 | 7 | 0.000179 | 0.008466 | 0.428571 |
| membranous septum morphogenesis | GO:0003149 | 4 | 10 | 1.68E-05 | 0.001235 | 0.4 |
| cellular response to L-glutamate | GO:1905232 | 2 | 5 | 0.002752 | 0.054002 | 0.4 |
| negative regulation of synaptic plasticity | GO:0031914 | 2 | 5 | 0.002752 | 0.054002 | 0.4 |
| beta-2 adrenergic receptor binding | GO:0031698 | 2 | 5 | 0.002752 | 0.054002 | 0.4 |
| positive regulation of basement  membrane assembly involved in  embryonic body morphogenesis | GO:1904261 | 2 | 5 | 0.002752 | 0.054002 | 0.4 |
| glial cell migration | GO:0008347 | 3 | 9 | 0.000322 | 0.012379 | 0.333333 |
| cerebellar granule cell precursor  proliferation | GO:0021930 | 2 | 6 | 0.003641 | 0.064307 | 0.333333 |
| negative regulation of histone H3-K4 methylation | GO:0051572 | 2 | 6 | 0.003641 | 0.064307 | 0.333333 |
| negative regulation of type B pancreatic cell apoptotic process | GO:2000675 | 2 | 6 | 0.003641 | 0.064307 | 0.333333 |
| telencephalon regionalization | GO:0021978 | 2 | 6 | 0.003641 | 0.064307 | 0.333333 |
| contractile ring | GO:0070938 | 2 | 6 | 0.003641 | 0.064307 | 0.333333 |
| neurotransmitter reuptake | GO:0098810 | 2 | 6 | 0.003641 | 0.064307 | 0.333333 |
| Wnt signaling pathway involved in  midbrain dopaminergic neuron  differentiation | GO:1904953 | 2 | 6 | 0.003641 | 0.064307 | 0.333333 |
| modulation by virus of host process | GO:0019048 | 2 | 7 | 0.004646 | 0.073253 | 0.285714 |
| negative regulation of thymocyte  apoptotic process | GO:0070244 | 2 | 7 | 0.004646 | 0.073253 | 0.285714 |
| axonemal microtubule | GO:0005879 | 2 | 7 | 0.004646 | 0.073253 | 0.285714 |
| regulation of odontogenesis of  dentin-containing tooth | GO:0042487 | 2 | 7 | 0.004646 | 0.073253 | 0.285714 |
| adrenergic receptor signaling pathway | GO:0071875 | 2 | 7 | 0.004646 | 0.073253 | 0.285714 |
| cardiac atrium morphogenesis | GO:0003209 | 2 | 7 | 0.004646 | 0.073253 | 0.285714 |
| K48-linked polyubiquitin modification-dependent protein binding | GO:0036435 | 2 | 8 | 0.005762 | 0.082511 | 0.25 |
| bone trabecula formation | GO:0060346 | 2 | 8 | 0.005762 | 0.082511 | 0.25 |
| pharyngeal arch artery morphogenesis | GO:0061626 | 2 | 8 | 0.005762 | 0.082511 | 0.25 |
| positive regulation of transcription from RNA polymerase II promoter involved in cellular response to chemical stimulus | GO:1901522 | 2 | 8 | 0.005762 | 0.082511 | 0.25 |
| ion homeostasis | GO:0050801 | 2 | 8 | 0.005762 | 0.082511 | 0.25 |
| peptidyl-lysine deacetylation | GO:0034983 | 2 | 8 | 0.005762 | 0.082511 | 0.25 |
| type II transforming growth factor beta (TGF-beta) receptor binding | GO:0005114 | 2 | 8 | 0.005762 | 0.082511 | 0.25 |
| positive regulation of ion transmembrane transporter activity | GO:0032414 | 2 | 8 | 0.005762 | 0.082511 | 0.25 |
| vesicle targeting | GO:0006903 | 2 | 8 | 0.005762 | 0.082511 | 0.25 |
| necroptotic signaling pathway | GO:0097527 | 2 | 8 | 0.005762 | 0.082511 | 0.25 |
| negative regulation of norepinephrine  secretion | GO:0010700 | 2 | 8 | 0.005762 | 0.082511 | 0.25 |
| positive regulation of epidermal cell  differentiation | GO:0045606 | 2 | 8 | 0.005762 | 0.082511 | 0.25 |
| hepatocyte differentiation | GO:0070365 | 3 | 13 | 0.000793 | 0.024137 | 0.230769 |
| cardiac epithelial to mesenchymal  transition | GO:0060317 | 3 | 13 | 0.000793 | 0.024137 | 0.230769 |
| cellular response to cholesterol | GO:0071397 | 4 | 18 | 0.000114 | 0.006561 | 0.222222 |
| leading edge membrane | GO:0031256 | 2 | 9 | 0.006989 | 0.089435 | 0.222222 |
| heparin biosynthetic process | GO:0030210 | 2 | 9 | 0.006989 | 0.089435 | 0.222222 |
| somatic stem cell division | GO:0048103 | 2 | 9 | 0.006989 | 0.089435 | 0.222222 |
| negative regulation of cell division | GO:0051782 | 2 | 9 | 0.006989 | 0.089435 | 0.222222 |
| hemostasis | GO:0007599 | 2 | 9 | 0.006989 | 0.089435 | 0.222222 |
| astral microtubule organization | GO:0030953 | 2 | 9 | 0.006989 | 0.089435 | 0.222222 |
| regulation of type I interferon-mediated signaling pathway | GO:0060338 | 2 | 9 | 0.006989 | 0.089435 | 0.222222 |
| chondroitin sulfate proteoglycan  biosynthetic process | GO:0050650 | 2 | 9 | 0.006989 | 0.089435 | 0.222222 |
| glomerulus development | GO:0032835 | 2 | 9 | 0.006989 | 0.089435 | 0.222222 |
| cAMP catabolic process | GO:0006198 | 2 | 9 | 0.006989 | 0.089435 | 0.222222 |
| peptidyl-tyrosine dephosphorylation  involved in inactivation of protein kinase activity | GO:1990264 | 2 | 9 | 0.006989 | 0.089435 | 0.222222 |
| cardiac muscle cell proliferation | GO:0060038 | 3 | 14 | 0.000954 | 0.026892 | 0.214286 |
| transforming growth factor beta receptor binding | GO:0005160 | 3 | 14 | 0.000954 | 0.026892 | 0.214286 |

**Table S6.** Biological processes enriched (ratio of no. genes upregulated/ no. of genes in the pathway >0.2; P≤0.05) with annotated genes that were upregulated specifically by the F7+niraparib treatment in OVCAR3.

| **Biological process** | **Gene  Ontology ID** | **No. genes upregulated** | **No. of genes in the pathway** | **P-Value** | **Corrected P-Value** | **Enrichment** |
| --- | --- | --- | --- | --- | --- | --- |
| regulation of hair cycle | GO:0042634 | 2 | 6 | 0.000346 | 0.012517 | 0.333333 |
| negative regulation of glucocorticoid receptor signaling pathway | GO:2000323 | 2 | 6 | 0.000346 | 0.012517 | 0.333333 |
| maternal process involved in parturition | GO:0060137 | 2 | 7 | 0.000444 | 0.015537 | 0.285714 |
| detoxification of copper ion | GO:0010273 | 4 | 16 | 7.09E-07 | 8.54E-05 | 0.25 |
| response to metal ion | GO:0010038 | 3 | 12 | 1.94E-05 | 0.000955 | 0.25 |
| response to molecule of bacterial origin | GO:0002237 | 2 | 8 | 0.000554 | 0.017807 | 0.25 |
| negative regulation of growth | GO:0045926 | 4 | 18 | 1.06E-06 | 0.000105 | 0.222222 |

**Table S7.** Biological processes enriched (ratio of no. genes downregulated/ no. of genes in the pathway >0.2; P≤0.05) with annotated genes that were downregulated specifically by the F7+niraparib treatment in OVCAR3.

| **Biological process** | **Gene  Ontology ID** | **No. genes  downregulated** | **No. of genes in the pathway** | **P-Value** | **Corrected P-Value** | **Enrichment** |
| --- | --- | --- | --- | --- | --- | --- |
| pronephros development | GO:0048793 | 2 | 6 | 0.000211 | 0.020349 | 0.333333 |
| positive regulation of gastrulation | GO:2000543 | 2 | 7 | 0.00027 | 0.020349 | 0.285714 |
| cellular hypotonic response | GO:0071476 | 2 | 7 | 0.00027 | 0.020349 | 0.285714 |
| positive regulation of phospholipase  activity | GO:0010518 | 2 | 7 | 0.00027 | 0.020349 | 0.285714 |
| negative regulation of potassium ion transmembrane transporter activity | GO:1901017 | 2 | 8 | 0.000337 | 0.022371 | 0.25 |
| centriolar subdistal appendage | GO:0120103 | 2 | 9 | 0.000411 | 0.022371 | 0.222222 |
| negative regulation of epithelial cell  differentiation | GO:0030857 | 2 | 9 | 0.000411 | 0.022371 | 0.222222 |
| negative regulation of sodium ion  transmembrane transporter activity | GO:2000650 | 2 | 9 | 0.000411 | 0.022371 | 0.222222 |

**Table S8.** Different letters signify quantitative PCR-based determination of mRNA steady-state levels that are significantly different from all combinations of pairs according to Tukey-Kramer honest significant difference (HSD; P ≤ 0.05). Quantitative PCR-based determination of the mRNA steady-state level in Caov3 and OVCAR3 cell lines was done at 6, 9 and 24 h of treatment with niraparib, F7, or a combination of niraparib+F7 relative to control (1.00% v/v methanol + 0.60% v/v DMSO) and is presented in Figure 2. Since Treatment x Time interaction was found to be significant by two-way ANOVA (p≤ 0.05), one-way ANOVA was performed for each time-point separately; (n = 3).

| Gene |  | Caov3 | | | | OVCAR3 | | | |
| --- | --- | --- | --- | --- | --- | --- | --- | --- | --- |
| *DDIT3* | Treatment/Time | 6h | 9h | 24h | 6h | | 9h | 24h |  |
|  | Control | C | c | *D* | B | | b | *A* |  |
|  | Niraparib | C | d | *C* | B | | b | *A* |  |
|  | F7 | A | a | *A* | A | | a | *A* |  |
|  | Niraparib+F7 | B | b | *B* | A | | a | *A* |  |
|  | Treatment x Time interaction | Significant p<0.0001 | | | Significant p<0.0001 | | | |  |
| *ATF4* | Treatment/Time | 6h | 9h | 24h | 6h | | 9h | 24h |  |
|  | Control | B | b | *C* | BC | | b | *B* |  |
|  | Niraparib | B | b | *C* | C | | b | *B* |  |
|  | F7 | A | a | *A* | A | | b | *A* |  |
|  | Niraparib+F7 | A | a | *B* | B | | a | *B* |  |
|  | Treatment x Time interaction | Significant p<.0004 | | | Significant p<.0001 | | | |  |
| *TRIB3* | Treatment/Time | 6h | 9h | 24h | 6h | | 9h | 24h |  |
|  | Control | C | c | *D* | B | | d | *D* |  |
|  | Niraparib | C | c | *C* | B | | c | *C* |  |
|  | F7 | A | a | *A* | A | | a | *B* |  |
|  | Niraparib+F7 | B | b | *B* | A | | b | *A* |  |
|  | Treatment x Time interaction | Significant p<0.0001 | | | Significant p<0.0001 | | | |  |

**Table S9.** Different letters signify quantitative PCR-based determination of mRNA steady-state levels that are significantly different from all combinations of pairs according to Tukey-Kramer honest significant difference (HSD; P ≤ 0.05). Quantitative PCR-based determination of the RNA steady-state level in Caov3 and OVCAR3 cell lines was done at 6, 9 and 24 h of treatment with niraparib, F7, or a combination of niraparib+F7 relative to control (1.00% v/v methanol + 0.60% v/v DMSO) and is presented in Figure 3. Since Treatment x Time interaction was found to be significant by two-way ANOVA (p≤ 0.05), one-way ANOVA was performed for each time-point separately. ; (n = 3).

| Gene |  | Caov3 | | | | OVCAR3 | | | |
| --- | --- | --- | --- | --- | --- | --- | --- | --- | --- |
| *CIP1* | Treatment/Time | 6h | 9h | 24h | 6h | | 9h | 24h |  |
|  | Control | B | c | *D* | B | | d | *B* |  |
|  | Niraparib | B | c | *B* | AB | | b | *A* |  |
|  | F7 | A | a | *C* | A | | c | *B* |  |
|  | Niraparib+F7 | A | b | *A* | A | | a | *A* |  |
|  | Treatment x Time interaction | Significant p<.0001 | | | Significant p<.0001 | | | |  |
| *PLK1* | Treatment/Time | 6h | 9h | 24h | 6h | | 9h | 24h |  |
|  | Control | A | a | *A* | A | | a | *A* |  |
|  | Niraparib | C | b | *C* | BC | | c | *C* |  |
|  | F7 | B | b | *B* | AB | | b | *B* |  |
|  | Niraparib+F7 | C | b | *C* | C | | bc | *C* |  |
|  | Treatment x Time interaction | Significant p<.0002 | | | Significant p<.0001 | | | |  |
| *INK4c* | Treatment/Time | 6h | 9h | 24h | 6h | | 9h | 24h |  |
|  | Control | A | a | *A* | A | | A | A |  |
|  | Niraparib | B | ab | *AB* | CD | | CD | B |  |
|  | F7 | B | c | *B* | C | | D | CD |  |
|  | Niraparib+F7 | B | bc | *B* | CD | | CD | CD |  |
|  | Treatment x Time interaction | Significant p<.4952 | | | Significant p<.0001 | | | |  |

**Data S1.** is provided separately, attached as an Excel file.
